# Dual fluorescence of tetraphenylethylene-substituted pyrenes with aggregation-induced emission characteristics for white-light emission†
†Electronic supplementary information (ESI) available: NMR, UV-vis and PL spectra, fluorescent photographs, CIE coordinates, and molecular orbital plots of TPE-Pys. CCDC 1837024 and 1837025. For ESI and crystallographic data in CIF or other electronic format see DOI: 10.1039/c8sc01709c


**DOI:** 10.1039/c8sc01709c

**Published:** 2018-05-31

**Authors:** Xing Feng, Chunxuan Qi, Hai-Tao Feng, Zheng Zhao, Herman H. Y. Sung, Ian D. Williams, Ryan T. K. Kwok, Jacky W. Y. Lam, Anjun Qin, Ben Zhong Tang

**Affiliations:** a Department of Chemistry , Hong Kong Branch of Chinese National Engineering Research Center for Tissue Restoration and Reconstruction , Institute for Advanced Study and Department of Chemical and Biological Engineering , The Hong Kong University of Science and Technology , Clear Water Bay , Kowloon , Hong Kong , China . Email: tangbenz@ust.hk; b NSFC Center for Luminescence from Molecular Aggregates , SCUT-HKUST Joint Research Laboratory , State Key Laboratory of Luminescent Materials and Devices , South China University of Technology , Guangzhou 510640 , P. R. China; c HKUST-Shenzhen Research Institute , No. 9 Yuexing 1st RD, South Area, Hi-tech ParkNanshan , Shenzhen 518057 , P. R. China

## Abstract

This article presents a new strategy to achieve white-light emission from single tetraphenylethylene-substituted pyrenes (TPE-Pys) with aggregation-induced emission (AIE) characteristics. TPE-Pys were synthesized by a Pd-catalyzed coupling reaction of a boronic acid or pinacol ester of pyrene and tetraphenylethylene (TPE) derivatives and showed multicolor emission by introducing different substituents on the phenyl rings of TPE. TPE-Pys with a TPE unit at the 1-position and asymmetric TPE units at 2,7-positions show dual fluorescence in THF/water mixtures to realize white-light emission with CIE coordinates of (*x* = 0.30 and *y* = 0.41) and (*x* = 0.21 and *y* = 0.16), respectively. The structure–property relationship of TPE-Pys were investigated to elucidate the origin of the white emission. The results showed that due to the weak electronic interaction of pyrene and its chromophoric units at the 2,7-positions and the constraint of the rotation of the TPE unit at the 1-position of pyrene, each component can exhibit its own emission color. The combination of appropriate colors gives rise to white-light emission. Such a principle of molecular design may open a new avenue for preparing advanced multicolor and multifunctional optical materials for organic electronics.

## Abstract 2

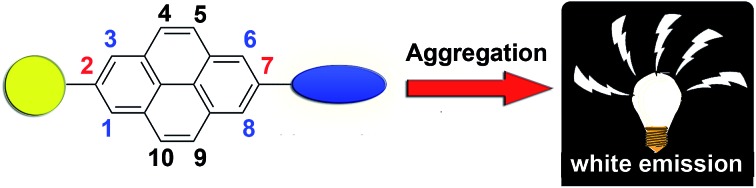
This article presents a new strategy to achieve white-light emission from single tetraphenylethylene-substituted pyrenes (TPE-Pys) with aggregation-induced emission (AIE) characteristics.

## Introduction

White organic light-emitting devices (WOLEDs) have attracted increasing attention both in academia and industry owing to their potential application in solid-state lighting, full-color displays and backlights for liquid crystalline displays.^1^–^4^ White light is a combination of multiple components with emission colours covering the entire visible range. Until now, most of the organic WOLEDs have been obtained by combining the emission from red, green and blue or blue and yellow emitters. However, such an approach requires complex device architectures and the resulting white emitters suffer from the drawbacks of phase segregation, colour aging and poor reproducibility to hamper their further development.^4^

Single molecular white-light-emitting compounds have attracted broad interest as they offer the possibility to achieve efficient and stable WOLEDs with even a simple single-layer device.^5^–^8^ Great effort has been made for the design of single molecular with white-light-emitting compounds in the past several years. By employing the techniques of monomer/excimer emission, excited-state intramolecular proton transfer,8*b* prompt/delayed dual fluorescence, and hybrid fluorescent/phosphorescent systems,^6^–^8^ luminophores with dual emission or single white-light emitters were generated. Despite these great achievements, simple molecular systems and straightforward designs to afford single white-light-emitters are still rare. Theoretically, different luminophoric units in a single molecule would exhibit their inherent emission color only if the energy/charge transfer and electron delocalization are suppressed.^9^ Unfortunately, these conditions are difficult to achieve in organic π-conjugated molecules. On the other hand, the use of single molecular white emitters in organic devices would simplify the fabrication process and lower the price to finally lead to high device robustness.

Pyrene belongs to the family of polycyclic aromatic hydrocarbons (PAHs) and emits deep blue emission with high quantum yield (QY) in dilute solutions. However, the fluorescence would be quenched when pyrene molecules are aggregated. Such a phenomenon of aggregation-caused quenching (ACQ)^10^ has limited its high-technological application.^11^ Thus, how to realize high-performance pyrene-based luminescent materials and how to suppress their ACQ effect are key issues in pyrene chemistry research. Aggregation-induced emission (AIE) is an abnormal photophysical phenomenon observed in some twisted molecules, such as tetraphenylethene (TPE) and siloles.^12^ AIE luminogens (AIEgens) show negligible emission in solution but enhanced emission in the solid state. The discovery of AIE has elementally solved the ACQ effect of traditional luminophores. Moreover, it has been demonstrated that the introduction of AIEgen to ACQ luminophores generates adducts with AIE characteristics. For example, 1,2,2-triphenyl-1-pyrenylethene and 1,2-diphenyl-1,2-dipyrenylethene show fantastic AIE characteristics with excimer emission in the aggregated state and crystal state.^13^ Mono-, di-, tri- and tetra-substituted pyrenes with TPE as the substituent at the 1-, 3-, 6- and 8-positions of pyrene or the K-region of the 4-position or 2,7-positions also emit intensely in the solid state.^14^ The AIE characteristics of these pyrene derivatives stem from their twisted structures due to the introduction of nonplanar arylvinyl units to prevent emission quenching in the solid state by detrimental π–π stacking interactions.

**Scheme 1:**
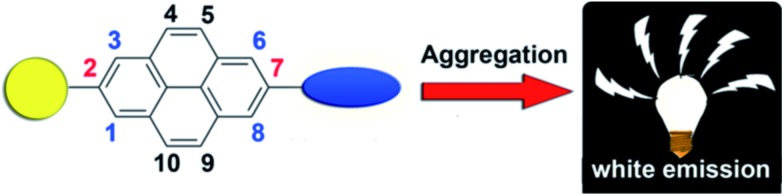
Molecular design of a pyrene-based white-emissive material.

Theoretically, as the nodal plane of pyrene passes through its carbon atoms at 2,7-positions in the HOMO and LUMO, substituents at these positions will interact weakly with the central core.^15^ For example, the uridine ring exhibited a weak electronic interaction with the pyrene core when it was substituted at the 2-position instead of the 1-position.^16^ Due to such a reason, as shown in Scheme 1, it is expected that white-light-emission may be achieved in pyrene derivatives by attaching chromophoric units with appropriate emission colors at the 2,7-positions.

According to our previous investigation, substituents at the *para*-positions of the phenyl rings of TPE exert a stronger influence on its emission. For instance, 1,1,2,2-tetrakis(*p*-ethoxylphenyl)ethene shows a tunable emission by topography changes.^17^ TPE with diethylamino groups, on the other hand, shows a yellow emission.^18^ Herein, we attached TPE unit(s) to pyrene with an attempt to realize white-light emission by molecular engineering. The structure–property relationship of the synthesized compounds were systematically investigated. The results show that TPE effectively suppresses the ACQ effect of pyrene to afford color-tunable adducts with AIE characteristics. Interestingly, except the asymmetric 2,7-disubstituted pyrene, the 1-substituted one shows also dual fluorescence in THF/water mixtures, which can be further tuned to white light by adjusting the water fraction. Such results provide a general strategy to achieve white emission from pyrene-based organic emitters.

## Results and discussion

### Synthesis and characterization

**Scheme 2:**
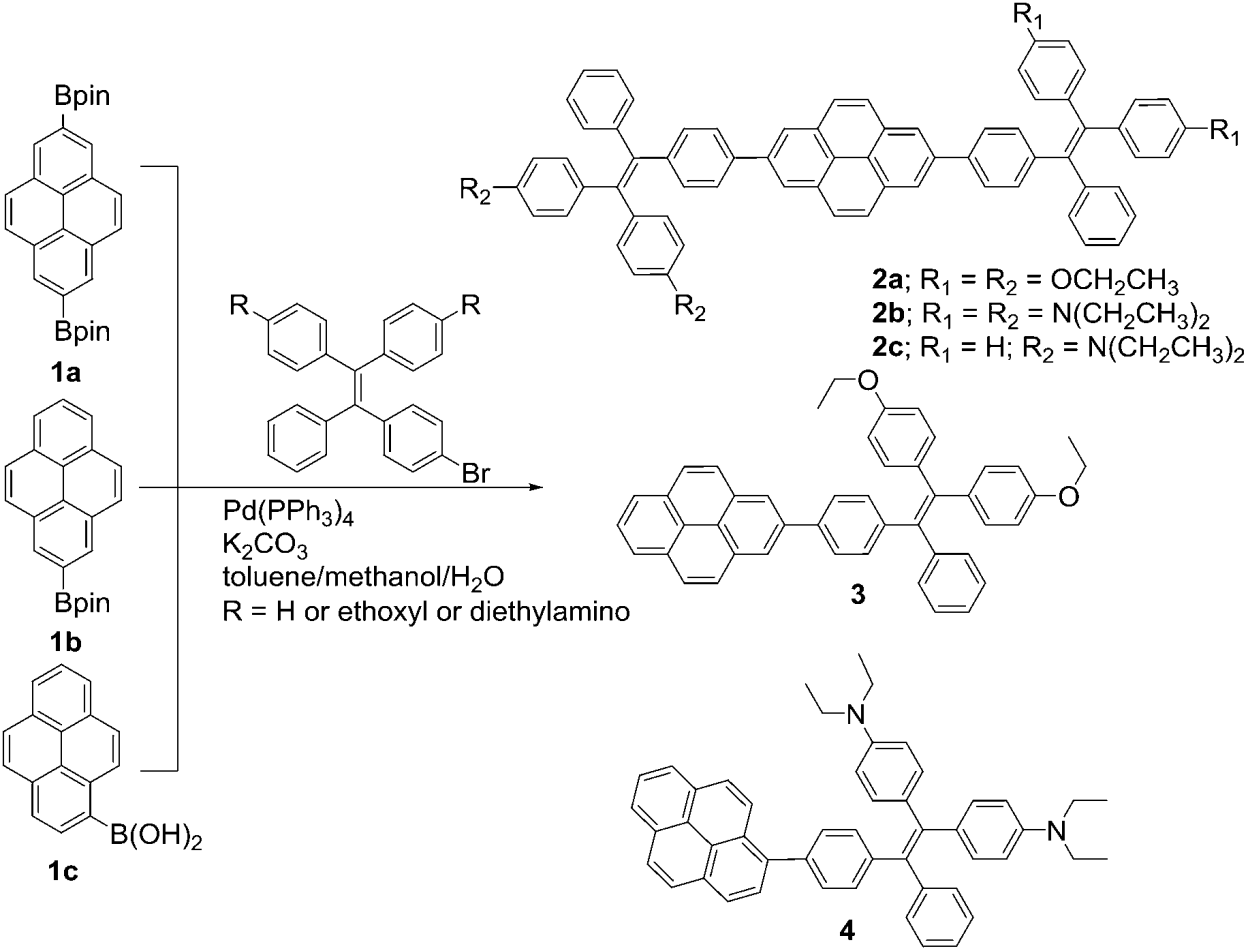
Synthetic route to TPE-Pys.

TPE-Pys were synthesized by Pd-catalyzed coupling of monobromo-TPE derivatives with 2,7-bis(4,4,5,5-tetramethyl-1,3,2-dioxaborolan-2-yl)pyrene (**1a**), 4,4,5,5-tetramethyl-2-(pyren-2-yl)-1,3,2-dioxaborolane (**1b**) and 4,4,5,5-tetramethyl-2-(pyren-1-yl)-1,3,2-dioxaborolane (**1c**), respectively (Scheme 2). The detailed procedures are given in the Experimental section at the end of the manuscript. All the compounds were characterized by ^1^H/^13^C NMR and high-resolution mass spectroscopies with satisfactory results (see Fig. S1–S10 in the ESI†). They exhibit good solubility in common organic solvents such as dichloromethane, *N*,*N*-dimethylformamide, tetrahydrofuran and dimethylsulfoxide, but dissolve fairly in aqueous solutions.

**Fig. 1:**
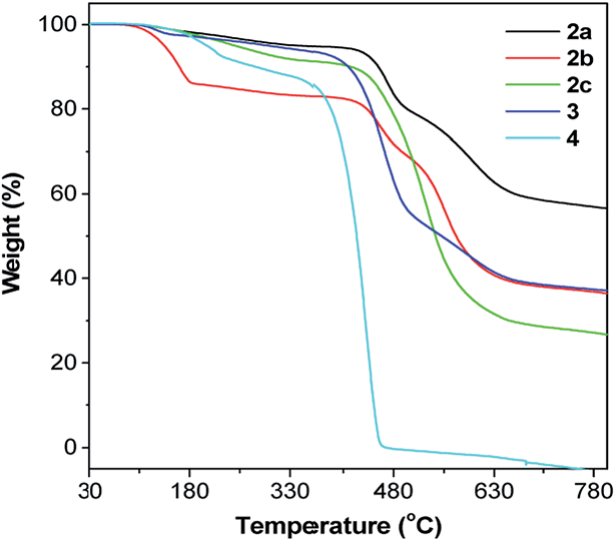
TGA curves of TPE-Pys recorded under nitrogen at a heating rate of 10 °C min^–1^.

The thermal properties of TPE-Pys were investigated by thermogravimetric analysis (TGA) and differential scanning calorimetry (DSC). As shown in Fig. 1, all the compounds show high thermal stability and degrade at high temperature (421 °C for **2a**, 422 °C for **2c**, 388 °C for **3** and 355 °C for **4**). The linear 2,7-disubstituted pyrenes **2a–c** are thermally more stable than the mono-substituted ones **3** and **4**. It is noteworthy that compound **2b** loses about 15% of its weight at 180 °C, possibly due to the evaporation of the solvent trapped inside. Afterwards, the curve remains flat until the molecule starts to degrade at 420 °C. The amount of carbonized residue (char yield) after heating to 800 °C of **2a–c** and **3** was higher (60% for **2a**, 40% for **2b** and **3** and 30% for **2c**) than that of **4** due to their higher aromatic content.^19^ No signals related to the glass transition temperatures were detected in the DSC thermograms of TPE-Pys. The melting points of 2-substituted and 2,7-disubstituted compounds **2** and **3** fall at around 150–190 °C (Table 1) and are higher than that of their 1-substituted counterpart **4** (142 °C). Both the TGA and DSC data suggest that the number of substituents and substitution positions affect the thermal properties.

**Table 1:** Physical properties of TPE-Pys^*a*^

Cpd	*λ*_abs_ (nm)	*λ*_em_ (nm) sol/aggre/film	*Φ*_F_ (%) sol/film	*T*_m_ (°C)	*T*_d_ (°C)
**2a**	293, 343	462/498/474	0.8/46.7	153	421
**2b**	293, 304, 394	500/536/566	0.6/3.8	160	420
**2c**	284, 342, 392	435/436, 538/533	0.6/6.8	198	422
**3**	279, 342	441/493/502	0.5/19.8	142	388
**4**	278, 341, 400	386, 407, 429/386, 407, 429, 538/530	0.8/9.7	162	355

^*a*^Abbreviations: *λ*_abs_ = absorption maximum measured in THF at room temperature, *λ*_em_ = emission maximum in THF (sol), THF/water mixtures (1 : 99, v/v; aggre) and the solid state (film), *Φ*_F_ = fluorescence quantum yield, *T*_m_ = melting point determined by DSC and *T*_d_ = degradation temperature determined by TGA.

### Crystal structure analysis

**Fig. 2:**
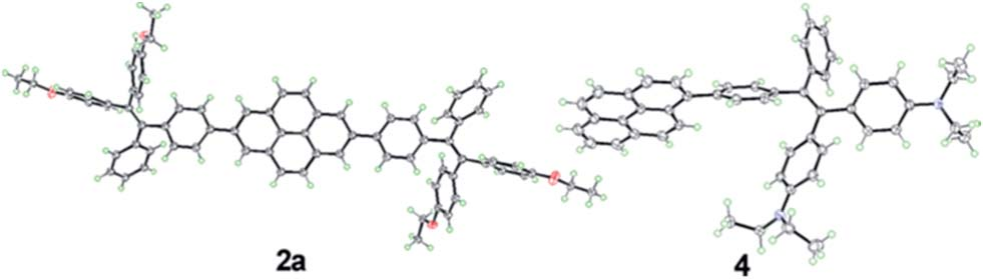
ORTEP drawings of **2a** and **4** with displacement ellipsoids drawn at the 50% probability level. Solvent molecules are omitted for clarity.

Crystals of **2a** and **4** with suitable quality for X-ray diffraction were cultivated by solvent diffusion of hexane into their concentrated THF solutions at room temperature. The crystal data are summarized in Table 2 and the ORTEP drawings are illustrated in Fig. 2. Both **2a** and **4** crystallize in a triclinic crystal system with the *P*1[combining macron] space group. The asymmetric unit cell of **2a** contains four molecules with CHCl_3_ and a disordered hexane molecule, while **4** contains two CHCl_3_ molecules without extra crystallized solvent molecules.

**Table 2:** Summary of crystal data of **2a** and **4**

Parameter	**2a**	**4**
Empirical formula	C_154_H_136_Cl_4_O_12_	C_50_H_46_N_2_
Formula weight [g mol^–1^]	2320.43	674.89
Crystal system	Triclinic	Triclinic
Space group	*P*1[combining macron]	*P*1[combining macron]
*a* [Å]	13.850(3)	10.1039(4)
*b* [Å]	15.150(3)	14.5087(9)
*c* [Å]	17.460(3)	15.0844(8)
*α* [°]	73.28(3)	65.241(5)
*β* [°]	78.26(3)	89.939(4)
*γ* [°]	77.43(3)	69.932(5)
Volume [Å^3^]	3385.5(12)	1859.55(19)
*Z*	1	2
Density, calcd [g m^–3^]	1.138	1.205
Temperature [K]	153(2)	99.97(10)
Unique reflns	20 341	10 336
Obsd reflns	12 996	6588
Parameters	743	469
*R*_int_	0.0976	0.0172
*R*[*I* > 2*σ*(*I*)]^*a*^	0.0755	0.1066
w*R*[*I* > 2*σ*(*I*)]^*b*^	0.2271	0.1143
GOF on *F*^2^	1.004	1.004

^*a*^Conventional *R* on *F*_hkl_: ∑||*F*_o_| – |*F*_c_||/*σ*|*F*_o_|.

^*b*^Weighted *R* on |*F*_*hkl*_|^2^: ∑[w(*F*_o_^2^ – *F*_c_^2^)^2^]/∑[w(*F*_o_^2^)^2^]^1/2^.

**Fig. 3:**
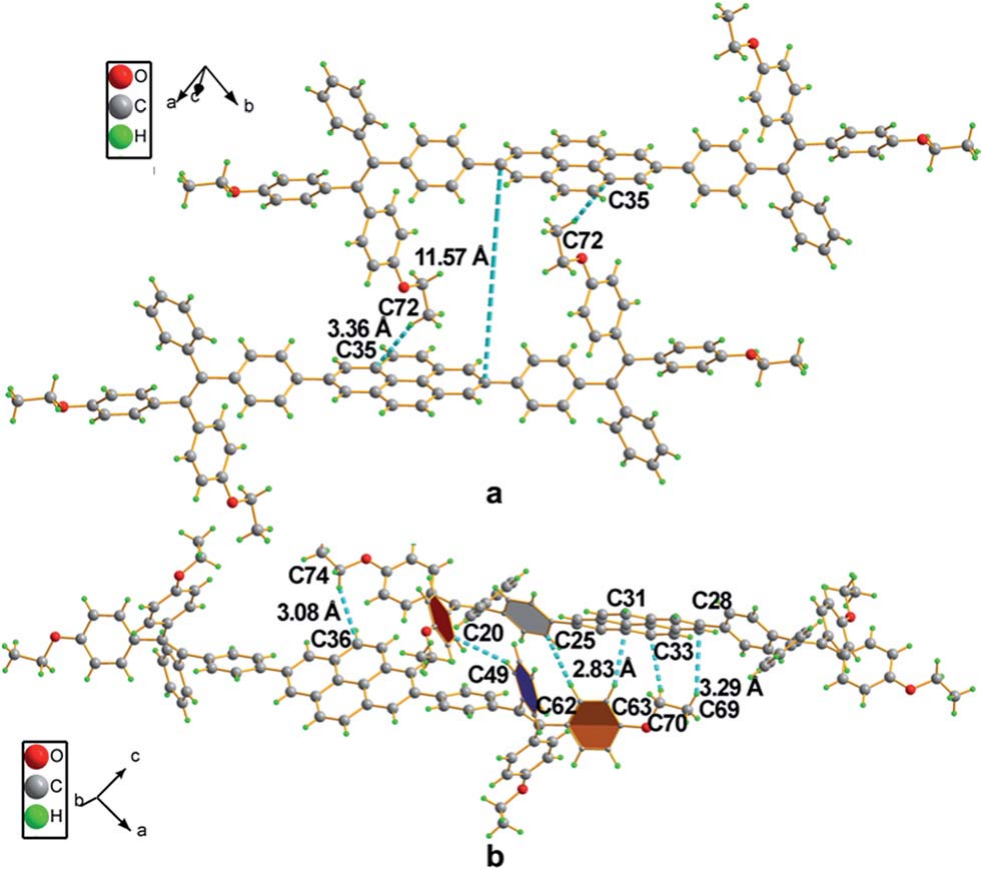
Packing arrangements in a single crystal of **2a**: (a) face-to-face patterns of pyrene moieties separated by TPE and (b) multiple weak C–H···π interactions between adjacent molecules with distances ranging from 3.08 Å to 3.29 Å.

The single crystal of **2a** exhibits two different packing patterns in an asymmetric cell unit. The four phenyl rings of TPE and the pyrene core are not located on the same plane but are arranged at considerable twisting angles. For example, the torsion angle between the pyrene core and the phenyl ring of TPE is 29.79°. As shown in Fig. 3a, the adjacent pyrene cores are separated by a long distance of 11.57 Å. This prevents their π–π stacking to quench the light emission. Multiple C–H···π interactions are formed in the crystal lattice (Fig. 3b), which effectively restrict the phenyl rings of TPE from undergoing intramolecular motions. The nonradiative decay pathways are blocked to allow **2a** to emit intense light in the aggregated state.

**Fig. 4:**
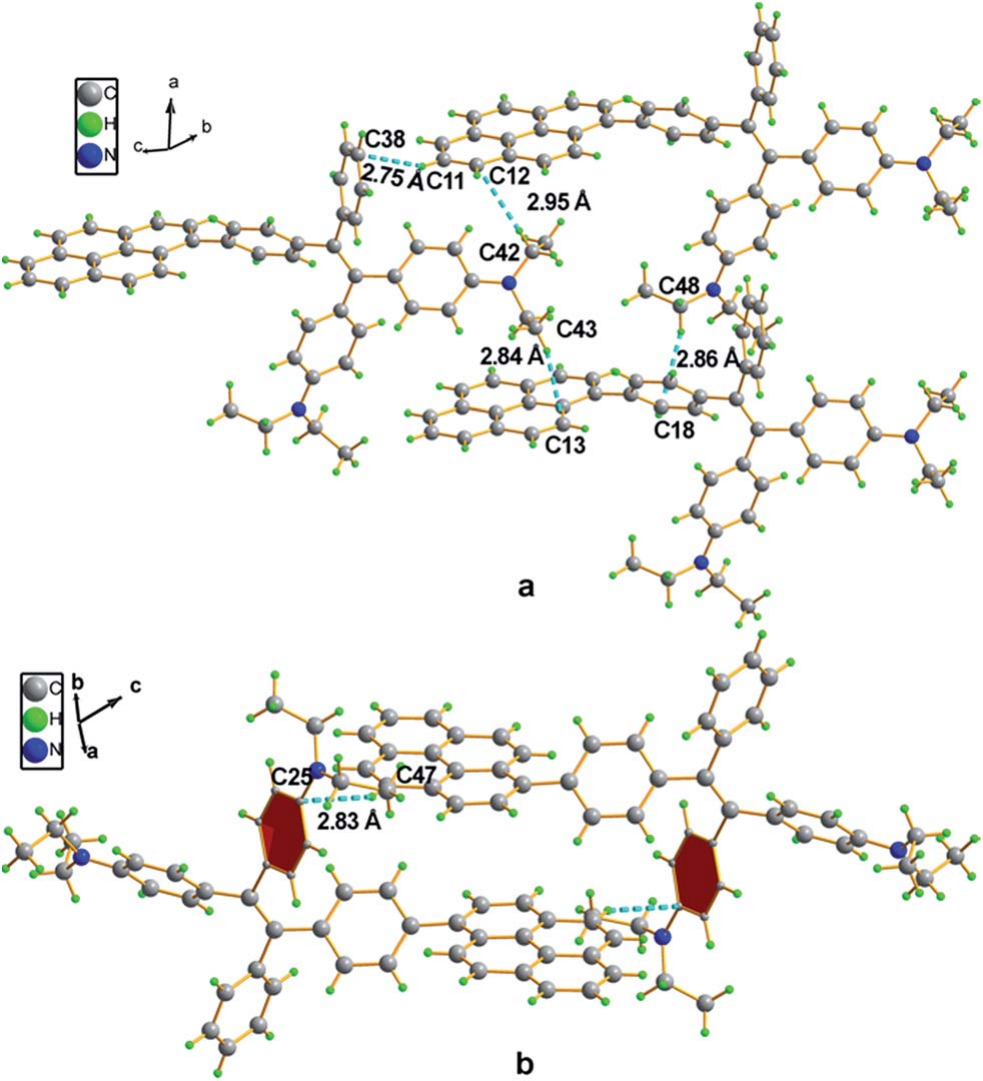
Representation of packing arrangements in a single crystal of **4**: (a) face-to-face patterns of pyrene moieties separated by TPE units and (b) head-to-tail packing mode with multiple C–H···π interactions with a distance of 2.83 Å.

Compound **4** adopts a twisted conformation similar to that of **2a** and the torsion angle between the terminal pyrene ring and the benzene ring of TPE is up to 48.6° (Fig. 4). The molecular motion of the TPE moiety is also suppressed by multiple intramolecular C–H···π interactions with distances of 2.75–2.95 Å. The pyrene rings are arranged in a head-to-tail fashion and are separated by the TPE units with a large distance of 8.08 Å. π–π stacking is less likely to occur to contribute enhanced fluorescence of the molecule in the solid state.

### Photophysical properties

Incorporation of different TPE units to the 1-, 2- or 2,7-positions of pyrene would affect its electronic and optical properties both in solution and solid states.17b,^20^ Thus, the absorption and photoluminescence (PL) of pyrene **2–4** were measured in different solvents (10 μM) at room temperature. Fig. S11–S16† show the spectra and Table S1† summarizes the results.

**Fig. 5:**
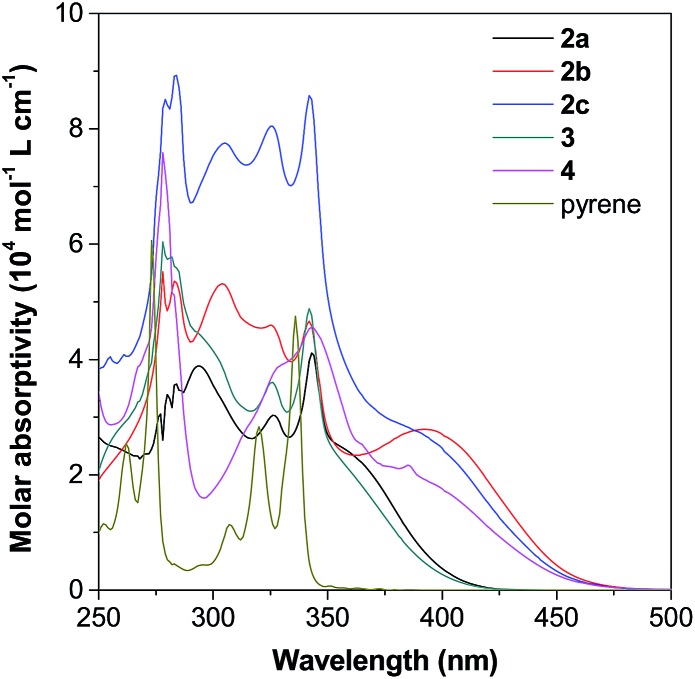
UV-vis spectra of **2–4** and pyrene in THF solutions (10 μM).

As shown in Fig. 5 as an example, the UV-vis spectra of **2–4** in THF show a strong absorption band at around 250–350 nm with a shoulder peak at around 350–450 nm. Compared with the spectrum of pyrene given in Fig. S11A,† the peaks at short wavelengths originate from the red-shift of the absorption of the pyrene unit, while the shoulder absorption one is ascribed to the intramolecular charge transfer between the pyrene and TPE units. According to Marder's report,17*c* 2,7-disubstituted pyrenes show a little influence on the S_2_ ← S_0_ transition but a large influence on the S_1_ ← S_0_ absorption. In contrast, the substituent at the 1-position affects both S_2_ ← S_0_ and S_1_ ← S_0_ transitions. Indeed, compound **4** shows a quite different absorption behaviour compared to its 2,7-disubstituted due to the different substitution position effect. The absorption of 2-disubstituted and 2,7-disubstituted pyrenes carrying the same TPE unit (**2b** and **2c**) and (**2a** and **3**) is very similar. This indicates the limited electronic communication of pyrene with the TPE unit(s) at these positions.

### AIE properties

**Fig. 6:**
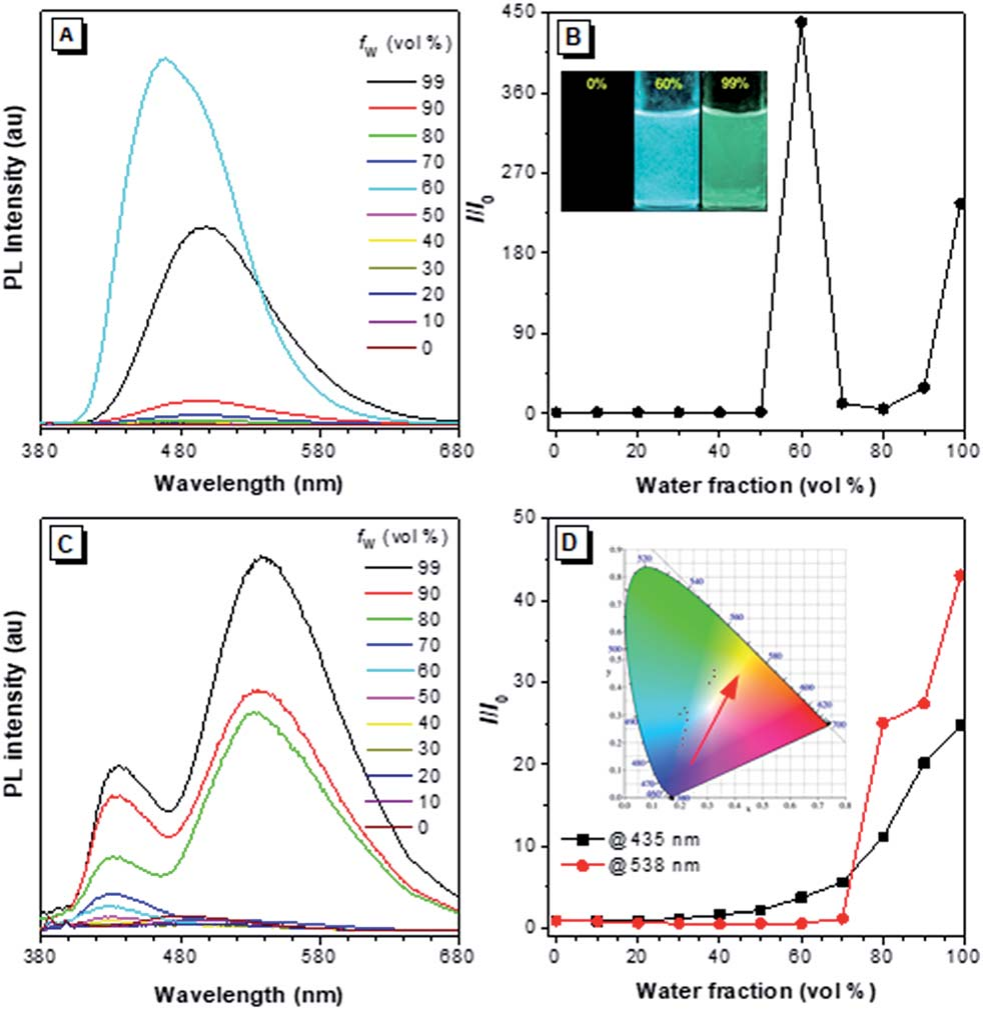
(A) PL spectra of **2a** in THF/water mixtures with different water fractions (*f*_w_). Excitation wavelength: 365 nm. (B) Plot of relative PL intensity (*I*/*I*_0_) *versus* the composition of a THF/water mixture of **2a**, where *I*_0_ is the PL intensity in pure THF solution. Inset: fluorescent photographs of **2a** in THF/water mixtures (*f*_w_ = 0, 60% and 99%) taken under UV illumination. (C) PL spectra of **2c** in THF/water mixtures with different water fractions (*f*_w_). (D) Plot of *I*/*I*_0_*versus* the composition of a THF/water mixture of **2c**, where *I*_0_ is the PL intensity in pure THF solution at 435 nm or 538 nm. Inset: CIE coordinates of the emission of **2c** in THF/water mixtures with different *f*_w_ (0–99 vol%).

The PL of **2–4** in dilute THF solutions and THF/H_2_O mixtures were investigated and the spectra are given in Fig. 6, S17–S19 and S22.† Traditional pyrene derivatives generally exhibit high PL intensity in dilute solutions but dim fluorescence in concentrated solutions or the aggregated state due to the ACQ effect. TPE-Pys **2a**, **2b** and **3** show no emission in pure THF solution but emit intensely in the aggregated state. Take **2a** as an example, its PL intensity at 462 nm was gradually enhanced upon addition of water to its THF solutions due to the aggregate formation. The higher the water fraction (*f*_w_), the stronger the PL intensity. At *f*_w_ = 60%, the PL intensity is 400-fold higher than that of pure THF solution. Further increasing the *f*_w_ leads to emission annihilation first but enhancement afterward. The PL maximum also gradually red-shifts to 498 nm. A possible explanation for such fluorescence changes is that different molecular aggregates of **2a** are formed in the presence of different *f*_w_.^21^ For example, crystalline aggregates may form at *f*_w_ = 60%, but in the presence of a large amount of water, the molecules of **2a** may cluster quickly to form amorphous aggregates. Analysis by powder XRD shows that different aggregates are formed at a *f*_w_ of 60% and 99% (Fig. S20†). Similar AIE behaviour was observed in **2b** (Fig. S17 and S21†), and the enhanced emission in the crystalline state compared to that in the amorphous state was ascribed to tighter molecular packing to restrict the molecular motion to block further the nonradiative relaxation process.^22^ Compound **3** is also AIE-active. Its PL intensity in THF solution is slightly increased upon water addition and the PL maximum is located at 493 nm in the THF/water mixture with *f*_w_ = 99% (Fig. S18†).^12^

Generally, pyrenes with the same substituents at 2,7-positions show a single emission color, while those derivatives with asymmetric 2,7-substituents give dual emission. The dual emission was in accordance with the limited electronic communication of substituents at 2,7-positions with the pyrene ring. As shown in Fig. 6c, compound **2c** emits a weak blue emission at 435 nm in dilute THF solution. A new emission peak appears at 538 nm when 70% of water is added to the THF solution. The yellow and blue emissions of the aggregates are ascribed to the weak electronic interaction of the pyrene core with the diethylamino-substituted TPE and the naked TPE fragment, respectively. Consequently, a warm white emission (CIE of *x* = 0.30, *y* = 0.41) with considerable QY (*Φ*_F_ = 12%) was observed by adjusting the ratio of THF to water to 8 : 2. During the entire process of addition of water, the emission color was tuned from blue to warm white and then to yellow (Fig. S19†).

Interestingly, when a diethylamino-substituted TPE unit was attached to the 1-position of pyrene, fantastic fluorescence was observed in the resulting compound **4**. As shown in Fig. S22,† the PL spectrum of **4** exhibits emission peaks at 386 nm, 407 nm, and 429 nm in dilute THF solution contributed mainly by the pyrene unit. Such a phenomenon suggests the inefficient molecular motion of the TPE unit due to its steric hindrance by the pyrene unit. Upon aggregate formation by water addition, a new peak at around 530 nm appears to result in a dual fluorescence and a cool white light with a CIE of *x* = 0.21, *y* = 0.16 and *Φ*_F_ of 22%. The fluorescence lifetimes of **2–4** are in the range of 2.12–4.50 ns in solution. On the other hand, all the compounds show more efficient light emission in the solid state with higher *Φ*_F_ (Table 1). Their PL spectra, however, exhibit only a single peak centered at 474 nm for **2a**, 566 nm for **2b**, 533 nm for **2c**, 502 nm for **3** and 530 nm for **4** (Fig. S23†), largely due to the energy transfer as the chromophoric units get further closer in the solid state.^23^

### Electrochemical properties

**Fig. 7:**
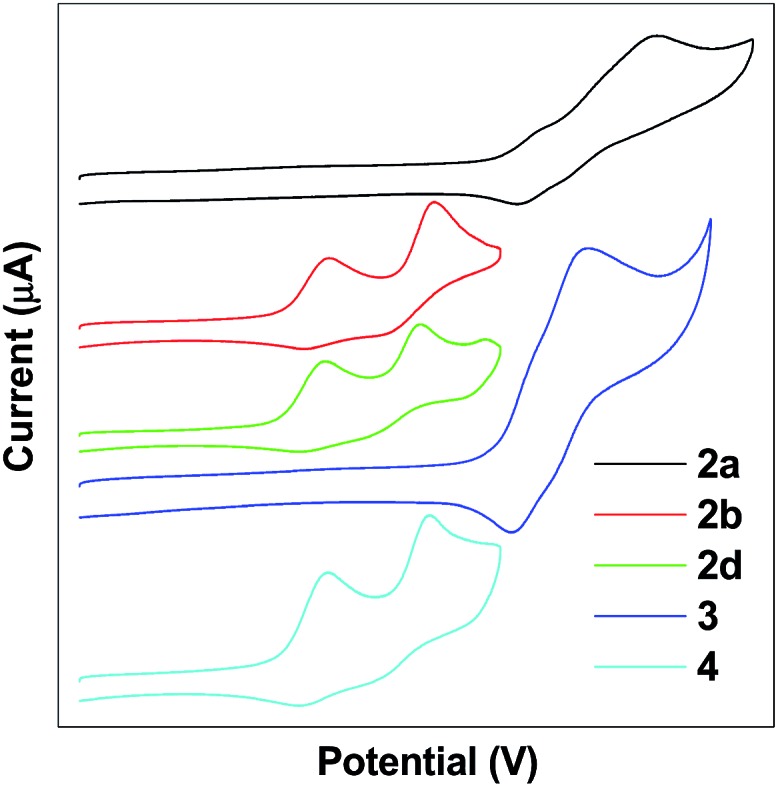
Cyclic voltammograms of **2**, **3** and **4**.

The electrochemical properties of TPE-Pys were studied by cyclic voltammetry in freshly distilled acetonitrile solution using 0.1 M tetrabutylammonium hexafluorophosphate as the electrolyte. Compounds **2a** and **3** display a quasi-reversible oxidation couple at 1.08 eV and 1.07 eV, and an irreversible oxidation wave at 1.37 eV and 1.20 eV (Fig. 7). Compounds **2b**, **2c** and **4** show two quasi-reversible oxidation processes at similar potentials (the first oxidation potential is located at 0.59 eV for **2b**, 0.58 eV for **2c** and 0.57 eV for **4**, and the second oxidation potential is found at 0.84 eV, 0.80 eV and 0.84 eV, respectively). The first oxidation wave was due to the electron-rich substituted TPE units and the second oxidation wave was associated with the whole conjugated molecular structure.

The HOMO levels are estimated from the first oxidation onset wave (*E*onsetox) by using the empirical formula HOMO = –*E*onsetox – 4.8. The energy gap (*E*_g_) was derived from the onset wavelength of the UV-vis spectrum and the corresponding LUMO levels are determined by the equation LUMO = HOMO + *E*_g_. The LUMO levels fall in the range of from –2.14 to –2.34 eV and the detailed electrochemical results are summarized in Table 3. Obviously, substituents on the TPE-units affect the HOMO level and the strong electron-donating diethylamino group helps to stabilize the HOMO energy.

**Table 3:** Electrochemical properties of TPE-Pys

Cpd	*λ*_onset_ (nm)^*a*^	*E*_onset_ (eV)^*b*^	LUMO (eV)^*c*^	HOMO (eV)^*c*^	*E*_g_ (eV)^*d*^
**2a**	402	0.99	–1.41(–2.34)	–4.87(–5.42)	3.46(3.08)
**2b**	455	0.48	–1.28(–2.18)	–4.38(–4.91)	3.10(2.72)
**2c**	448	0.48	–1.39(–2.14)	–4.44(–4.91)	3.05(2.77)
**3**	399	0.99	–1.41(–2.31)	–4.90(–5.42)	3.48(3.11)
**4**	451	0.48	–1.69(–2.16)	–4.63(–4.91)	2.94(2.75)

^*a*^ *λ*_onset_ = cut-off wavelength determined from the UV spectrum in CH_3_CN.

^*b*^Measured by CV in 0.1 M *n*-Bu_4_NPF_6_/CH_3_CN at a scan rate of 100 mV s^–1^.

^*c*^Calculated by DFT/B3LYP/6-31G* using Gaussian 03 with values given in the brackets determined by CV using the ferrocene HOMO level.

^*d*^Calculated from the empirical formula HOMO = –(4.8 + *E*onsetox – *E*onsetox (Fc)) with values calculated from the UV spectrum given in the brackets.

### Theoretical calculations

**Fig. 8:**
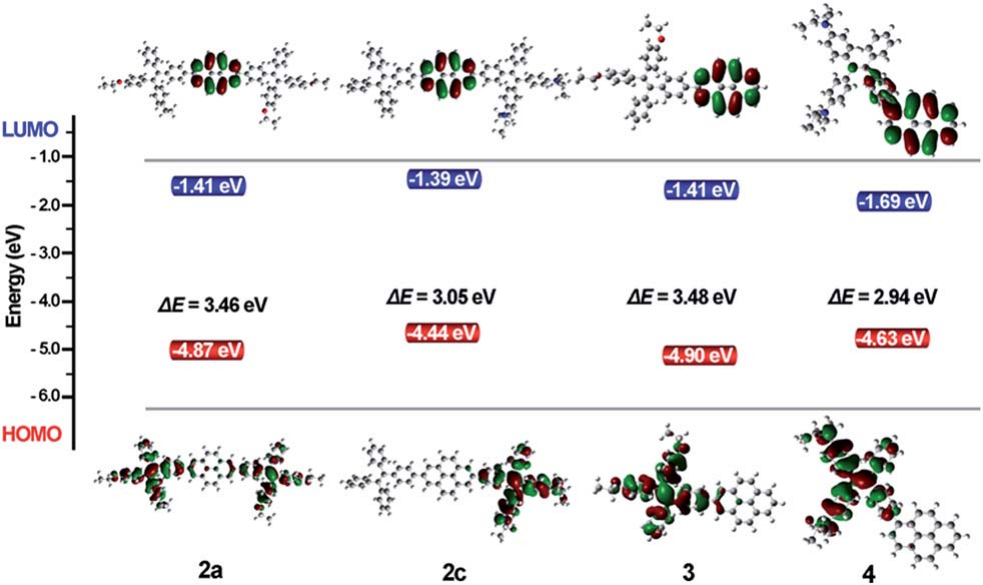
Molecular orbital plots of **2–4** calculated by the B3LYP/6-31G level.

To investigate the electron delocalization of TPE-Pys, DFT calculations were performed at the B3LYP/6-31G(d) level using the Gaussian 09 program. The optimized geometries and highest occupied molecular orbital (HOMO) and lowest unoccupied molecular orbital (LUMO) of **2–4** are presented in Fig. 8. All the TPE units of the molecules adopt a twisted conformation and the torsional angles between them and the pyrene core are 38° for **2** and **3**, and 56° for **4**. The HOMOs of all compounds **2–4** are localized on the TPE units, while those of LUMO levels are mainly localized on the central pyrene core (Fig. S24†). Basically, compared with its substituents, pyrene may act as an electron-donor or electron acceptor.^24^ In our case, because diethoxy- and diethylamino-substituted TPE units are electron-donating, the pyrene ring thus tends to serve as an acceptor.^25^ On the other hand, owing to the special electronic structure, weak electronic interactions between the pyrene core and the substituents at 2 or 2,7-positions were observed in **2** and **3**. In compound **4**, the TPE unit was linked to the 1-position of pyrene by the C–C bond to result in its larger orbital delocalization than **2** and **3**. Thus, it shows a lower *E*_g_ (2.94 eV) than **2** and **3** (*E*_g_ at 3.46 eV for **2a**, 3.10 eV for **2b**, 3.05 eV for **2c** and 3.48 eV for **3**, respectively). The theoretical calculation results are consistent with the CV data. Therefore, the substitution position of pyrene plays a significant role in adjusting the LUMO energy level, and the electronic effect of the TPE unit at 2,7-positions would affect the HOMO energy. Obviously, for compound **2**, the central pyrene core just plays a role in connecting the TPE units by the C–C single bond and weakly interacts with the peripheral substituents.

## Conclusions

In summary, based on the special electronic structure of pyrene, a series of pyrene-based luminogens **2–4** are facilely accessed by Suzuki coupling. These molecules exhibit tunable emission from blue (474 nm) to yellow (531 nm) both in solution and in the solid state. By introducing TPE units into pyrene, the resulting compounds exhibit AIE instead of ACQ characteristics with high thermal stability, strong fluorescence and high quantum yield in the solid state. In particular, TPE-Pys **2c** and **4** emit white-coloured fluorescence in THF/water mixtures. This is one of the rare examples of white-light emission achieved from a single AIE-active molecule *via* the control of its aggregated state emission by tuning the composition of the solvent mixture. This approach opens a door to the preparation of more attractive single molecules with white-light emission by AIE rational design.

## Experimental section

### General

^1^H and ^13^C NMR spectra (400 MHz) were recorded on a Bruker AV 400 spectrometer using chloroform-*d* solvent and tetramethylsilane as the internal reference. *J*-values are given in Hz. High-resolution mass spectra (HRMS) were recorded on a GCT premier CAB048 mass spectrometer operating in a MALDI-TOF mode. PL spectra were recorded on a Hitachi 4500 Spectrofluorometer. UV-vis absorption spectra were obtained on a Milton Ray Spectrofluorometer. PL quantum yields were measured using absolute methods. Cyclic voltammetry was carried out in 0.10 M tetrabutylammonium hexafluorophosphate in CH_3_CN at a scan rate of 100 mV s^–1^ at room temperature. Thermogravimetric analysis was carried on a TA TGA Q5500 under dry nitrogen at a heating rate of 10 °C min. Thermal transitions were investigated by differential scanning calorimetry using a TA DSC Q1000 under dry nitrogen at a heating rate of 10 °C min. The quantum chemistry calculation was performed on the Gaussian 03W (B3LYP/6-31G* basis set) software package.^26^ Crystallographic data of the compounds were collected on a Bruker APEX 2 CCD diffractometer with graphite monochromated Mo Kα radiation (*λ* = 0.71073 Å) in the *ω* scan mode.^27^,^28^ The structure was solved by charge flipping or direct method algorithms and refined by full-matrix least-squares methods on *F*^2^.^27^ All esds (except the esd in the dihedral angle between two l.s. planes) were estimated using the full covariance matrix. The cell esds were considered individually in the estimation of esds in distances, angles and torsion angles. Correlations between esds in cell parameters were only used when they were defined by crystal symmetry. An approximate (isotropic) treatment of cell esds was used for estimating esds involving l.s. planes. The final cell constants were determined through global refinement of the *xyz* centroids of the reflections harvested from the entire dataset. Structure solution and refinement were carried out using the SHELXTL-PLUS software package.^28^ The unit cell of **2a** containing two disordered dichloromethane molecules was taken into account in the SQUEEZE option of the PLATON program. Data (excluding structure factors) on the structures reported here had been deposited with the Cambridge Crystallographic Data Centre with deposition numbers. CCDC ; 1837024 and ; 1837025 contain the supplementary crystallographic data for this paper.†

### Materials

Unless otherwise stated, all other reagents used were purchased from commercial sources and were used without further purification. Tetrahydrofuran was distilled prior to use. Pyrene-2,7-diboronic acid bis(pinacol) ester was prepared as described previously.^29^

### Synthesis

The general procedure for the synthesis of TPEPys is given here. A mixture of 2,7-bis(4,4,5,5-tetramethyl-1,3,2-dioxaborolan-2-yl)pyrene (0.51 mmol, 1.0 eq.), corresponding bromo-substituted TPE (1.12 mmol, 2.2 eq.) in toluene (15 mL) and ethanol (4 mL) at room temperature was stirred under nitrogen. Then, potassium carbonate (2.04 mmol, 4.0 eq.) and tetrakis(triphenylphosphine)palladium (0.051 mmol, 0.1 eq.) were added. After the mixture was stirred for 30 min at room temperature under nitrogen, the mixture was heated to 90 °C for 48 h with stirring. After cooling to room temperature, the mixture was quenched with water, extracted with CH_2_Cl_2_ (3 × 100 mL), and washed with water and brine. The organic extracts were dried with MgSO_4_ and evaporated. The residue was purified by column chromatography using a CH_2_Cl_2_/hexane mixture as an eluent to give the target compound.

### Characteristic data

2,7-Bis{4-[2,2-bis(4-ethoxyphenyl)-1-phenylvinyl]phenyl}pyrene (**2a**). A light yellow solid of **2a** was obtained in 23% yield (120 mg) and a 20 mg by-product of 2-{4-[2,2-bis(4-ethoxyphenyl)-1-phenylvinyl]phenyl}pyrene **3** was obtained. ^1^H NMR (400 MHz, CDCl_3_), *δ* = 8.35 (s, 4H), 8.09 (s, 4H), 7.65 (d, *J* = 8.3 Hz, 4H), 7.20 (d, *J* = 8.3 Hz, 4H), 7.16–7.08 (m, 10H), 7.03 (d, *J* = 8.7 Hz, 4H), 6.97 (d, *J* = 8.7 Hz, 4H), 6.67 (d, *J* = 16.0, 8.8 Hz, 8H), 3.97 (d, *J* = 6.9, 1.7 Hz, 8H), 1.38 (d, *J* = 7.0, 4.2 Hz, 12H). ^13^C NMR (100 MHz, CDCl_3_), *δ* = 156.96, 143.74, 142.99, 139.93, 138.07, 137.98, 137.81, 135.64, 132.06, 132.03, 131.46, 130.91, 130.80, 127.23, 127.13, 126.47, 125.52, 123.07, 122.94, 113.02, 112.86, 76.72, 76.40, 76.09, 62.58, 14.23. HRMS (MALDI-TOF): *m*/*z* calcd for C_76_H_64_O_4_ 1040.4805; found 1038.4661 [M^+^].

2,7-Bis{4-[2,2-bis(4-diethylaminophenyl)-1-phenylvinyl]phenyl}pyrene (**2b**). A yellow solid of compound **2b** was obtained in 20% yield (130 mg). ^1^H NMR (400 MHz, CDCl_3_), *δ* = 8.38 (s, 4H), 8.09 (s, 4H), 7.67 (d, *J* = 8.2 Hz, 4H), 7.26 (d, *J* = 8.2 Hz, 4H), 7.18 (d, *J* = 4.3 Hz, 8H), 7.15–7.07 (m, 2H), 7.02 (d, *J* = 8.7 Hz, 4H), 6.96 (d, *J* = 8.7 Hz, 4H), 6.47 (d, *J* = 15.2, 8.8 Hz, 8H), 3.33 (q, *J* = 6.9 Hz, 16H), 1.15 (t, *J* = 7.0 Hz, 25H). ^13^C NMR (100 MHz, CDCl_3_), *δ* = 146.44, 146.35, 145.60, 144.98, 141.96, 138.70, 137.93, 135.63, 132.96, 132.92, 132.28, 131.77, 131.41, 131.29, 127.83, 127.62, 126.95, 125.44, 123.67, 123.53, 110.89, 110.78, 77.36, 77.25, 77.04, 76.72, 44.21, 12.68; HRMS (MALDI-TOF): *m*/*z* calcd for C_84_H_84_N_4_ 1148.6696; found 1146.6581 [M^+^].

2-[4-(Triphenylvinyl)phenyl]-7-{4-[2,2-bis(4-diethylaminophenyl)-1-phenylvinyl]phenyl} pyrene (**2c**). A mixture of pyrene-2,7-diboronic acid bis(pinacol) ester (0.51 mmol, 1.0 eq.), [2-(4-bromophenyl)ethene-1,1,2-triyl]tribenzene (0.56 mmol, 1.1 eq.), 1-(4-bromophenyl)-2-(4-diethylaminophenyl)-1,2-diphenylethene (0.56 mmol, 1.1 eq.) in toluene (15 mL) and ethanol (4 mL) at room temperature was stirred under argon. Then, potassium carbonate (1.02 mmol, 2.0 eq.) and tetrakis(triphenylphosphine)palladium (0.051 mmol, 0.1 eq.) were added. After the mixture was stirred for 30 min at room temperature under argon, the mixture was heated to 90 °C for 48 h with stirring. After cooling to room temperature, the mixture was quenched with water, extracted with CH_2_Cl_2_ (3 × 100 mL), and washed with water and brine. The organic extracts were dried with MgSO_4_ and evaporated. The residue was purified by column chromatography using a CH_2_Cl_2_/hexane mixture as the eluent to give a yellow solid 167 mg in 30% yield. ^1^H NMR (400 MHz, CDCl_3_), *δ* = 8.34 (d, *J* = 10.5 Hz, 4H), 8.06 (s, 4H), 7.65 (d, *J* = 8.1 Hz, 4H), 7.28–7.07 (m, 26H), 7.00 (d, *J* = 8.7 Hz, 2H), 6.94 (d, *J* = 8.6 Hz, 2H), 6.45 (d, *J* = 14.7, 8.8 Hz, 4H), 3.30 (q, *J* = 6.8 Hz, 8H), 1.13 (t, *J* = 6.7 Hz, 13H). ^13^C NMR (100 MHz, CDCl_3_), *δ* = 150.91, 145.79, 143.18, 137.60, 132.34, 132.30, 131.76, 131.70, 131.68, 131.66, 131.63, 131.56, 131.41, 131.13, 130.91, 130.86, 130.78, 130.67, 130.61, 127.29, 127.23, 127.12, 127.05, 127.00, 126.48, 126.32, 125.95, 122.95, 110.23, 110.12, 76.73, 76.41, 76.10, 43.56, 12.04. HRMS (MALDI-TOF): *m*/*z* calcd for C_76_H_64_O_4_ 1006.5226; found 1004.5077 [M^+^].

2-{4-[2,2-Bis(4-ethoxyphenyl)-1-phenylvinyl]phenyl}pyrene (**3**). A white solid of **3** was obtained 98 mg in 52% yield. ^1^H NMR (400 MHz, CDCl_3_), *δ* = 8.38 (d, *J* = 8.2 Hz, 3H), 8.10 (m, 3H), 7.89 (d, *J* = 7.2 Hz, 1H), 7.66 (d, *J* = 8.3 Hz, 2H), 7.57 (t, *J* = 7.7 Hz, 1H), 7.45 (t, *J* = 7.4 Hz, 1H), 7.22–7.11 (m, 7H), 7.04 (d, *J* = 8.7 Hz, 2H), 6.98 (d, *J* = 8.7 Hz, 2H), 6.69 (d, *J* = 8.7 Hz, 2H), 6.65 (d, *J* = 8.7 Hz, 2H). ^13^C NMR (100 MHz, CDCl_3_), *δ* = 156.96, 156.87, 143.74, 143.06, 140.91, 139.94, 138.27, 138.05, 137.97, 137.88, 135.64, 132.06, 132.03, 131.47, 130.90, 130.87, 128.36, 127.42, 127.33, 127.21, 127.13, 123.22, 123.16, 123.06, 123.00, 113.01, 112.87, 62.58, 14.23. HRMS (MALDI-TOF): *m*/*z* calcd for C_46_H_36_O_2_ 620.2715; found 620.2706 [M^+^].

1-{4-[2,2-Bis(4-diethylaminophenyl)-1-phenylvinyl]phenyl}pyrene (**4**). A mixture of pyrene-1-boronic acid (246 mg, 1 mmol, 1.0 eq.), 1-bromo-1-phenyl-2,2-bis(4-diethylaminophenyl)ethene (828 mg, 1.5 mmol, 1.5 eq.) in toluene (15 mL) and ethanol (4 mL) at room temperature was stirred under argon. Potassium carbonate (280 mg, 2 mmol, 2.0 eq.) and tetrakis(triphenylphosphine)palladium (59 mg, 0.051 mmol, 0.1 eq.) were added. After the mixture was stirred for 30 min at room temperature under argon, the mixture was heated to 90 °C for 48 h with stirring. After cooling to room temperature, the mixture was quenched with water, extracted with CH_2_Cl_2_ (3 × 100 mL) and washed with water and brine. The organic extracts were dried with MgSO_4_ and evaporated. The residue was purified by column chromatography using a CH_2_Cl_2_/hexane mixture as the eluent to give a yellow solid **4** in 68% yield (460 mg). ^1^H NMR (400 MHz, CDCl_3_), *δ* = 8.28–8.15 (m, 4H), 8.11 (s, 2H), 8.06–7.97 (m, 3H), 7.41 (d, *J* = 8.2 Hz, 2H), 7.25 (d, *J* = 17.5, 11.6, 5.1 Hz, 6H), 7.16 (d, *J* = 6.9 Hz, 1H), 7.06 (d, *J* = 8.8 Hz, 2H), 7.01 (d, *J* = 8.8 Hz, 2H), 6.56 (d, *J* = 8.8 Hz, 2H), 6.48 (d, *J* = 8.8 Hz, 2H), 3.36 (dt, *J* = 19.5, 7.0 Hz, 8H), 1.19 (dt, *J* = 17.3, 7.0 Hz, 12H). ^13^C NMR (100 MHz, CDCl_3_), *δ* = 146.39, 145.50, 144.76, 142.09, 138.11, 137.88, 135.85, 133.07, 132.93, 131.76, 131.59, 131.41, 131.04, 130.34, 129.81, 128.48, 127.67, 127.50, 127.17, 125.93, 125.60, 125.50, 124.96, 124.66, 110.88, 110.82, 77.37, 77.06, 76.74, 44.33, 44.22, 12.72, 12.69. HRMS (MALDI-TOF): *m*/*z* calcd for C_50_H_48_N_2_ 676.3817; found 674.3691 [M^+^].

## Conflicts of interest

There are no conflicts to declare.

## Supplementary Material

Supplementary information

Crystal structure data
