# Supplementary material for: Dual fluorescence of tetraphenylethylene-substituted pyrenes with aggregation-induced emission characteristics for white-light emission
Source: Chem Sci. 2018 May 31;9(25):5679–87. doi: 10.1039/c8sc01709c (PMC6050622; doi:10.1039/c8sc01709c)
Supplement: Supplementary file 1 [file SC-009-C8SC01709C-s001.pdf]

Electronic Supplementary Material (ESI) for Chemical Science.
This journal is © The Royal Society of Chemistry 2018

Supporting Information

Dual Fluorescence of Tetraphenylethylene-Substituted Pyrenes with Aggregation-Induced Emission Characteristics for White-Light Emission

Xing Feng,[‡a] Chunxuan Qi,[‡b] Hai-Tao Feng,[‡a] Zheng Zhao,[a] Herman H. Y. Sung,[a] Ian D. Williams,[a] Ryan T. K. Kwok,[a] Jacky W. Y. Lam,[a] Anjun Qin,[b] Ben Zhong Tang*[abc]

[a] Department of Chemistry, Hong Kong Branch of Chinese National Engineering Research Center for Tissue Restoration and Reconstruction, Institute for Advanced Study and Department of Chemical and Biological Engineering, The Hong Kong University of Science and Technology, Clear Water Bay, Kowloon, Hong Kong, China. E-mail: tangbenz@ust.hk

[b] NSFC Center for Luminescence from Molecular Aggregates, SCUT-HKUST Joint Research Laboratory, State Key Laboratory of Luminescent Materials and Devices, South China University of Technology, Guangzhou 510640, China

[c] HKUST-Shenzhen Research Institute, No. 9 Yuexing 1st RD, South Area, Hi-tech Park Nanshan, Shenzhen 518057, China

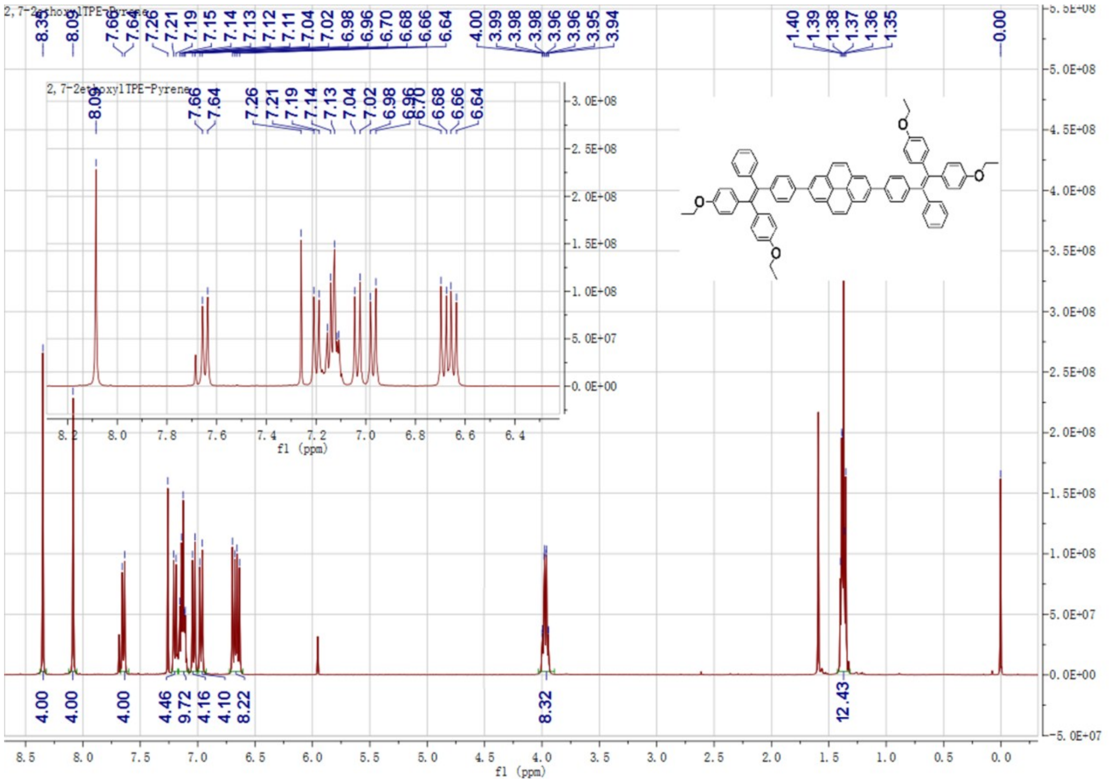

Fig. S1. ^{1}H NMR spectrum of **2a** in $CDCl_3$.

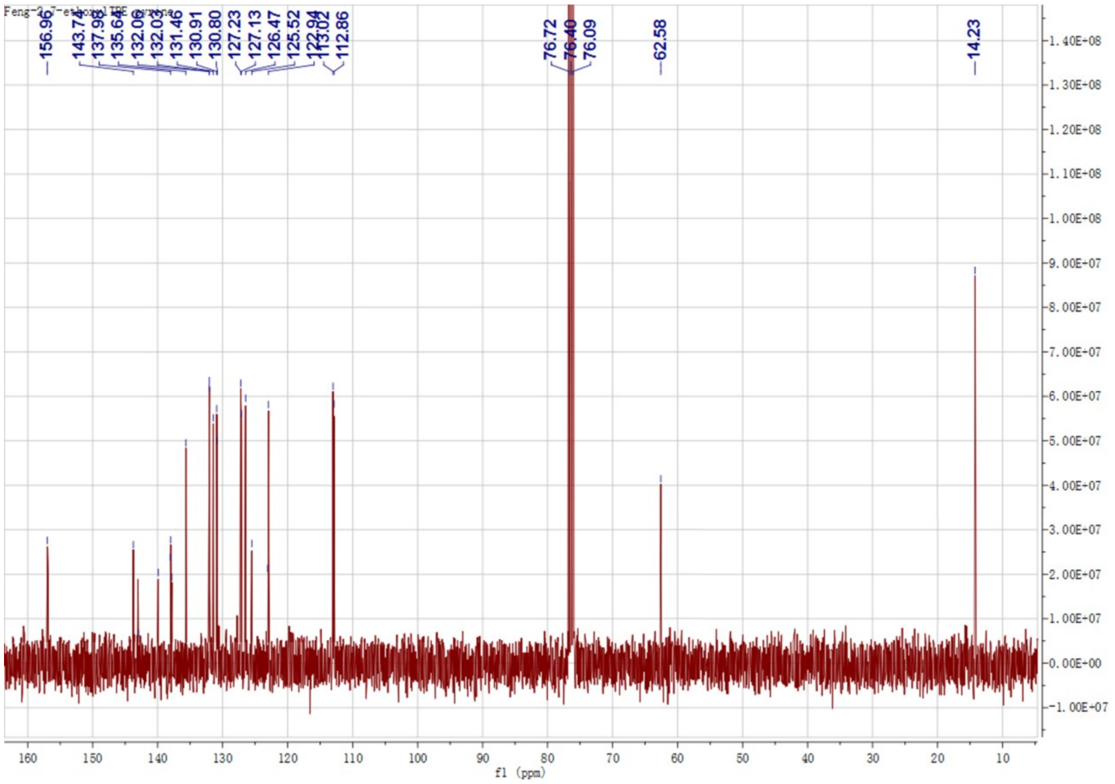

Fig. S2. ^{13}C NMR spectrum of **2a** in $CDCl_3$.

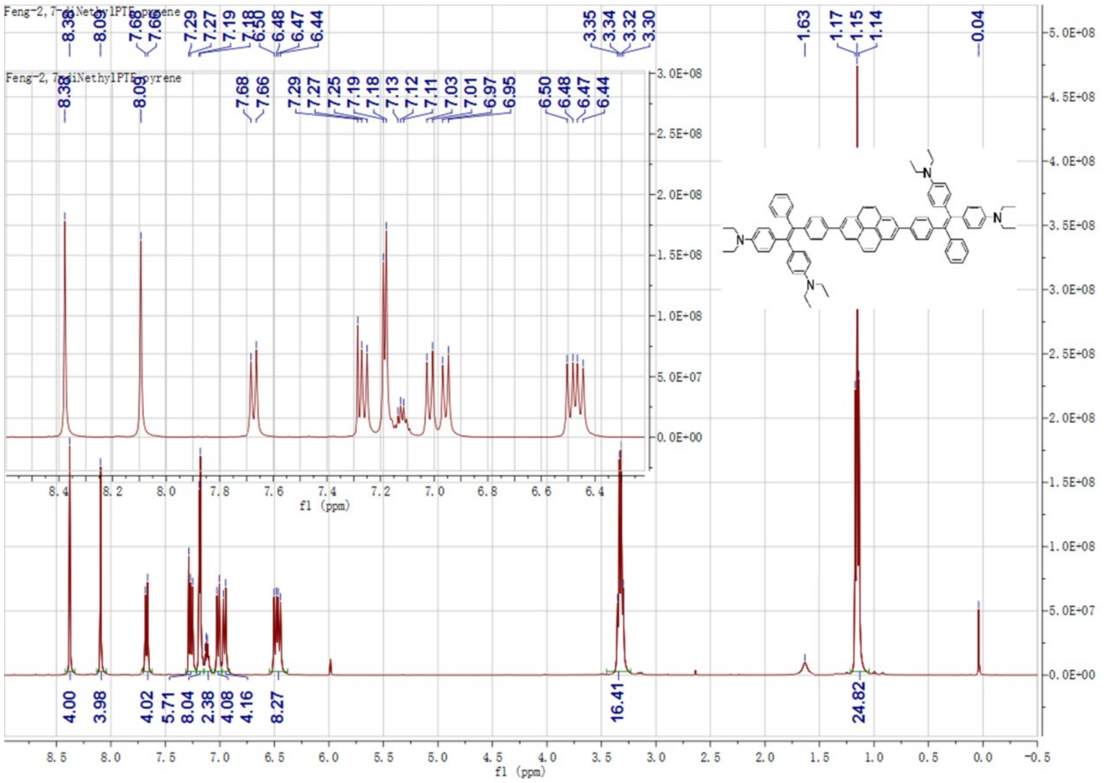

Fig. S3. ^{1}H NMR spectrum of **2b** in $CDCl_3$.

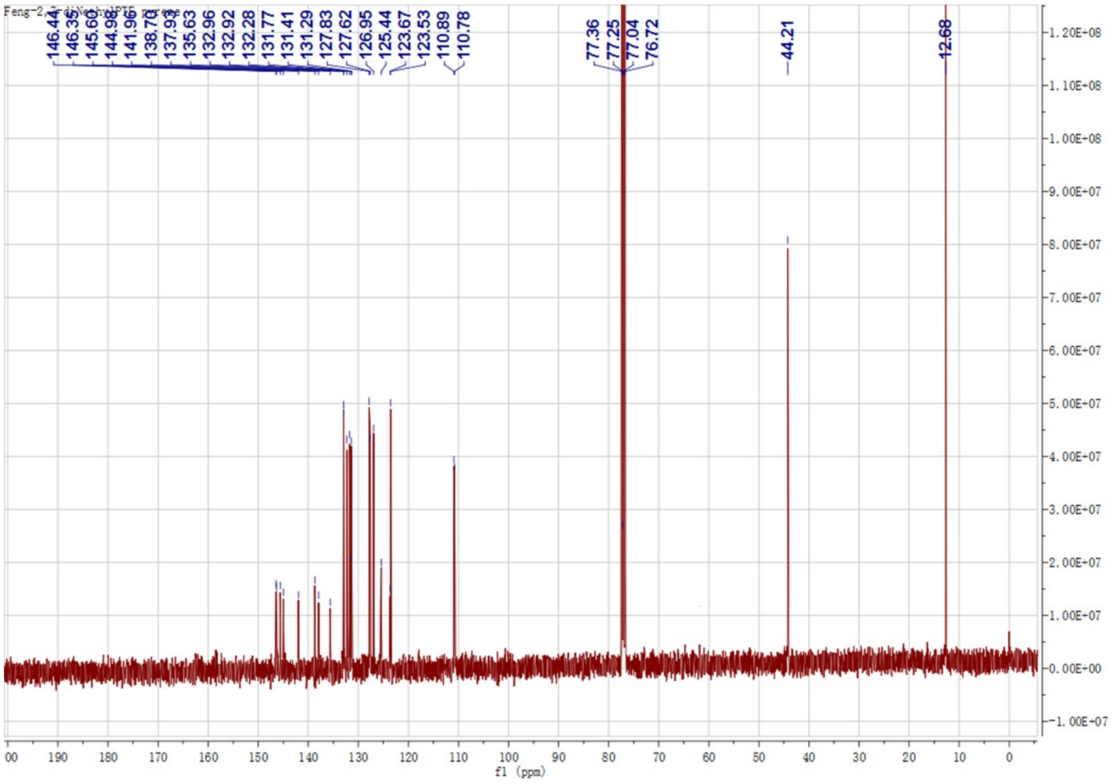

Fig. S4. ^{13}C NMR spectrum of **2b** in $CDCl_3$.

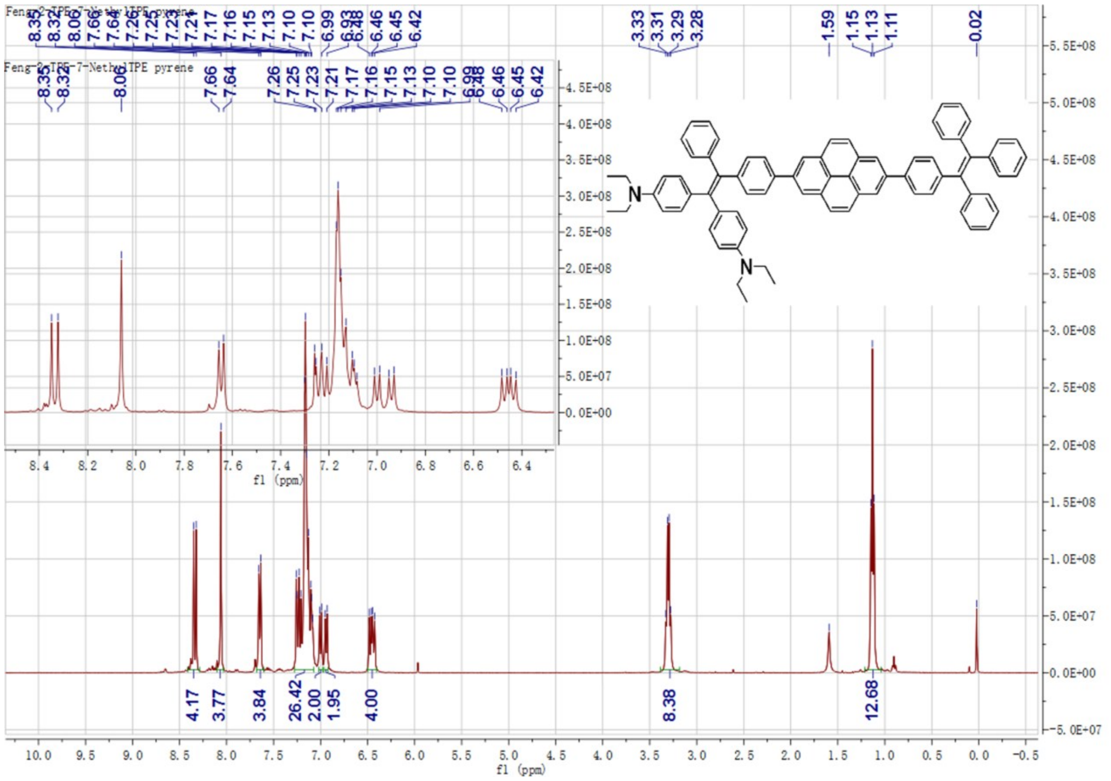

Fig. S5. ^{1}H NMR spectrum of **2c** in $CDCl_3$.

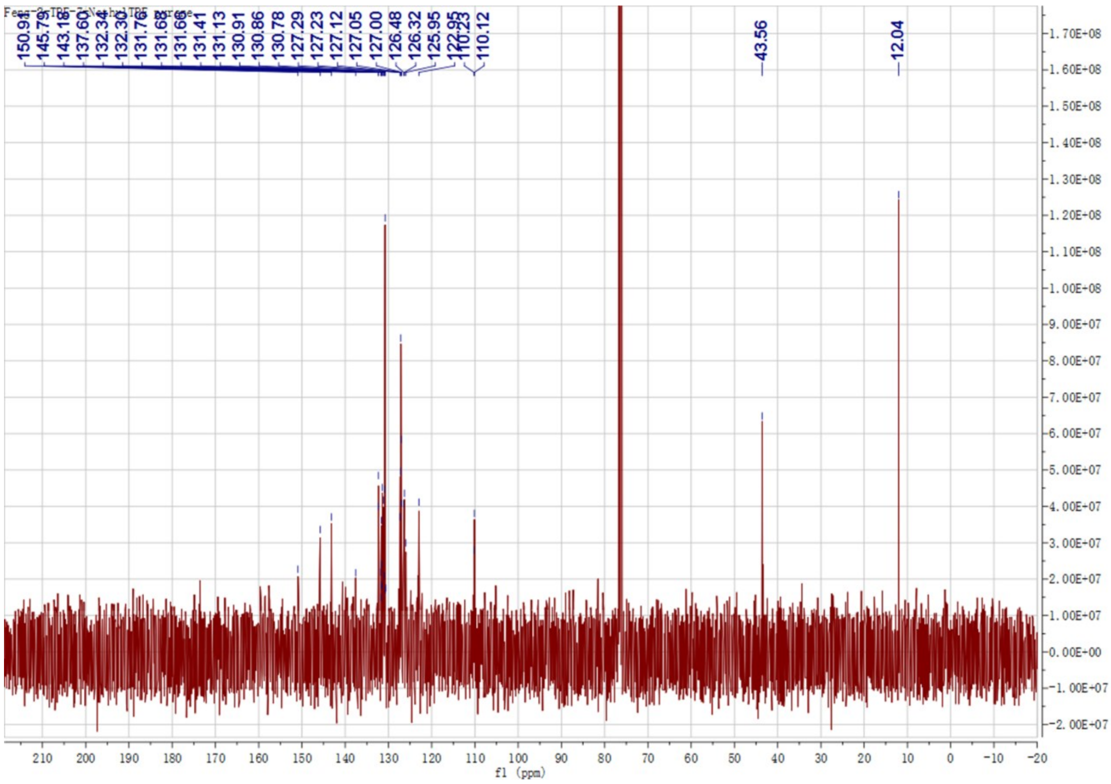

Fig. S6. ^{13}C NMR spectrum of **2c** in $CDCl_3$.

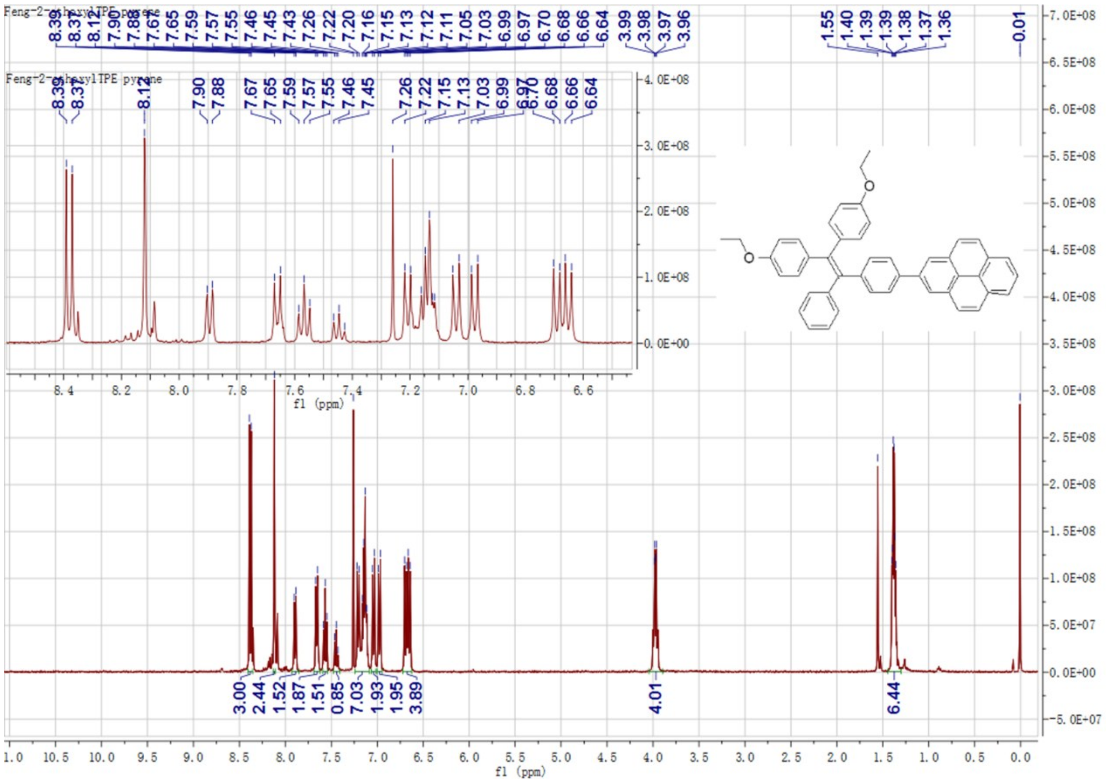

Fig. S7. ^{1}H NMR spectrum of **3** in $CDCl_3$.

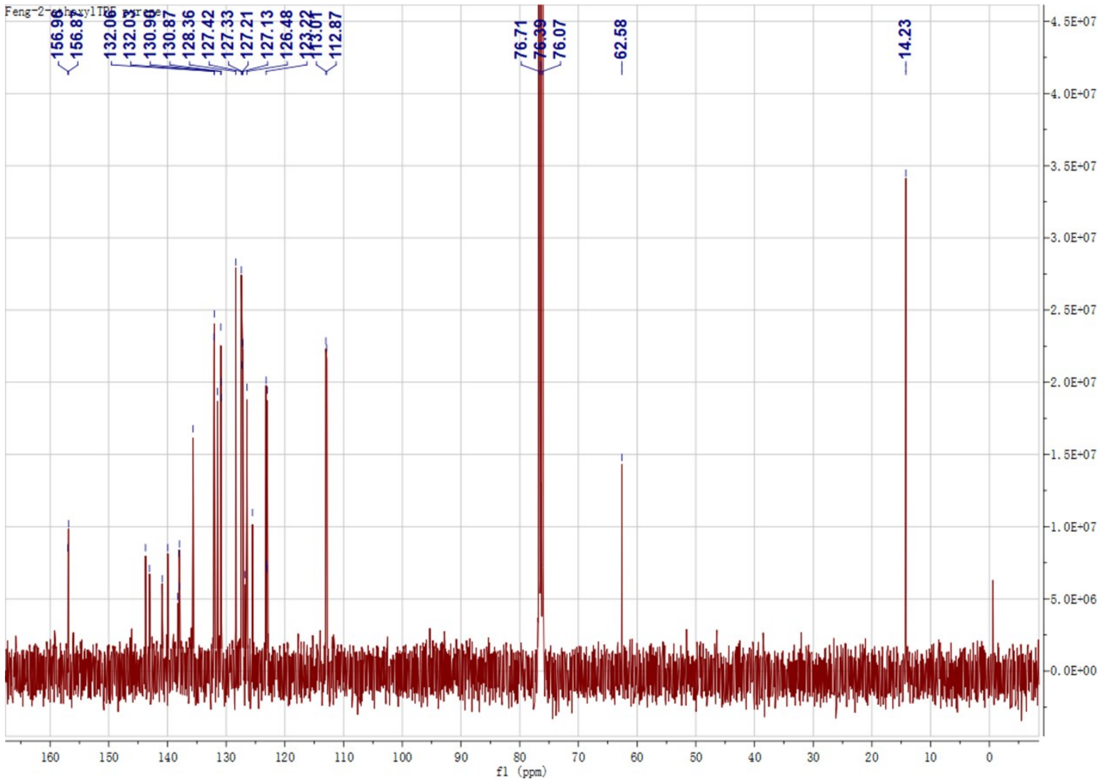

Fig. S8. ^{13}C NMR spectrum of **3** in $CDCl_3$.

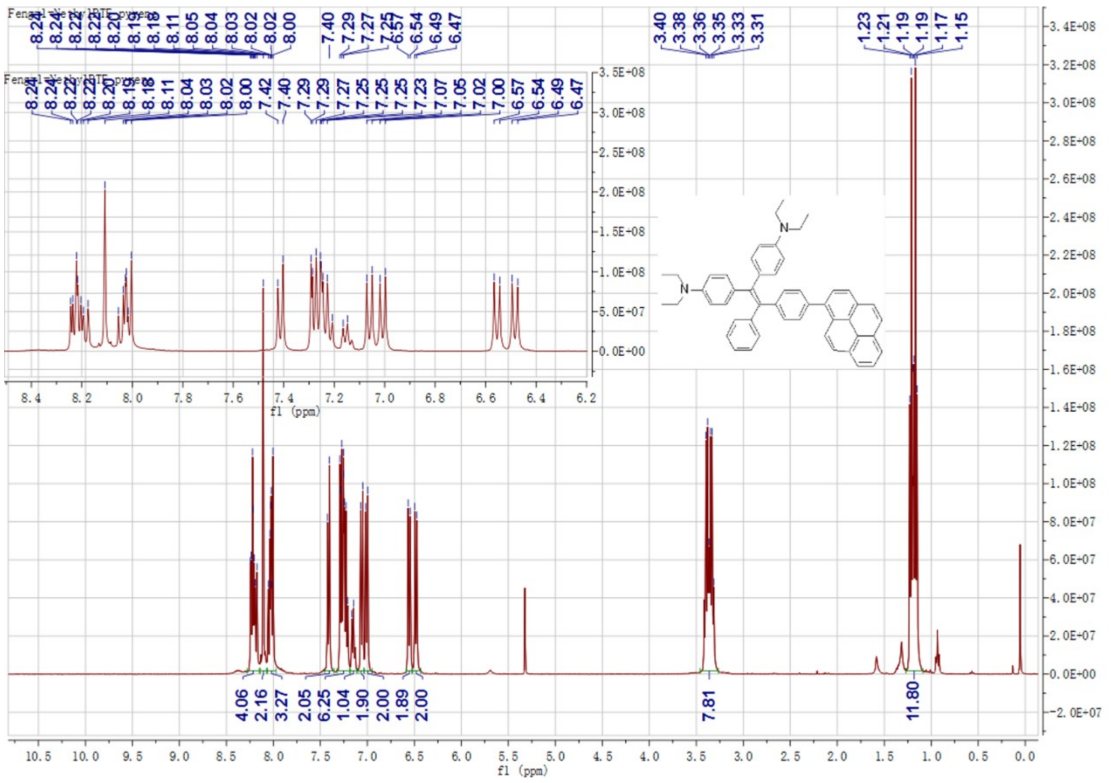

Fig. S9. ^{1}H NMR spectrum of **4** in $CDCl_3$.

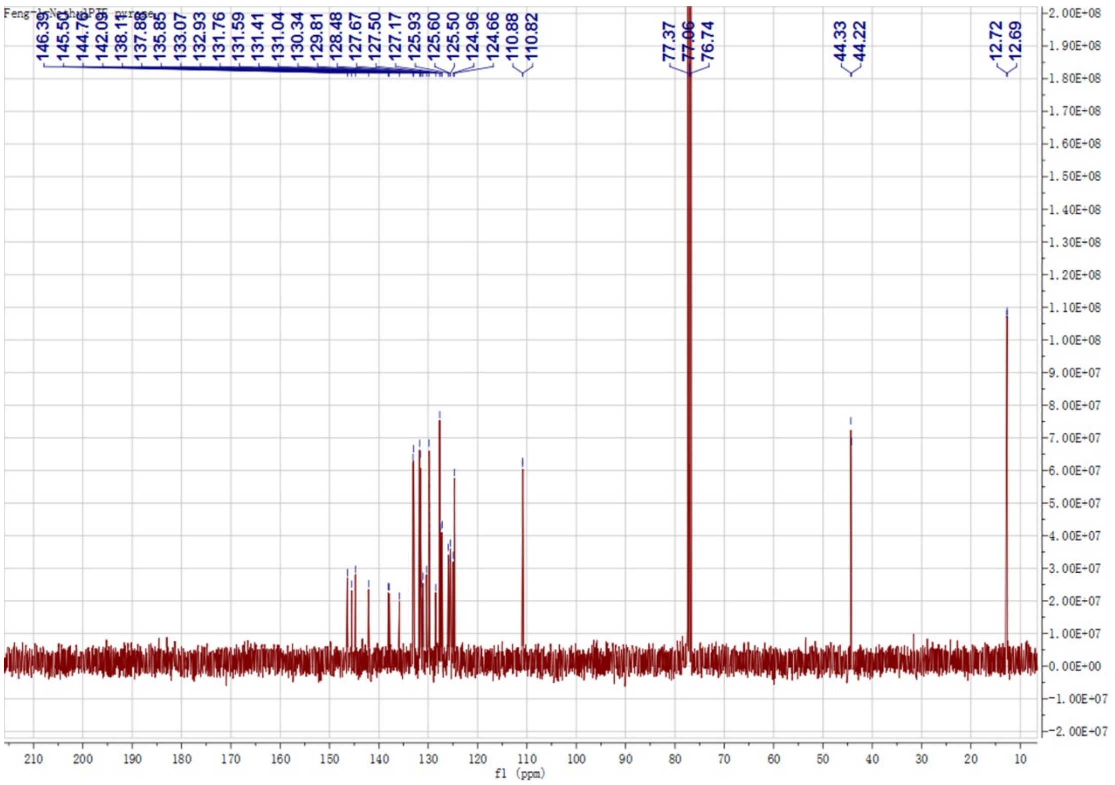

Fig. S10. ^{13}C NMR spectrum of **4** in $CDCl_3$.

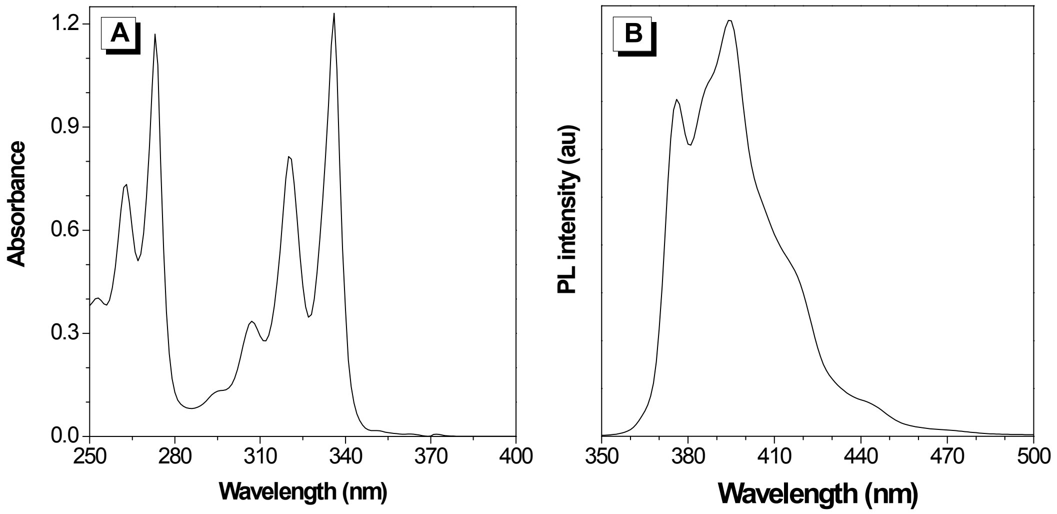

Fig. S11. (A) UV/Vis and (B) PL spectra of pyrene in THF (10 μM).

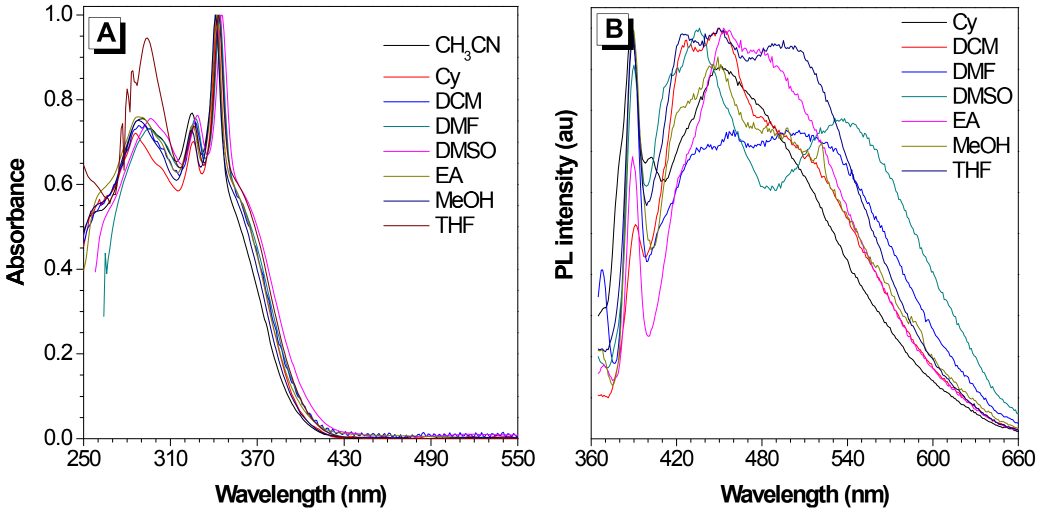

Fig. S12. (A) UV/Vis and (B) PL spectra of **2a** in different solvents (10 μM). λ_{ex} = 347 nm.

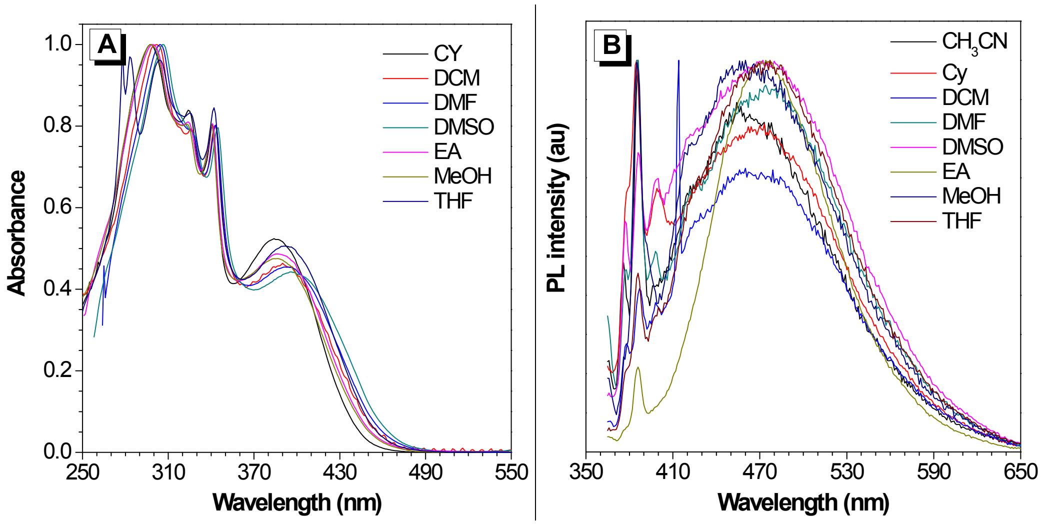

Fig. S13. (A) UV/Vis and (B) PL spectra of **2b** in different solvents (10 μM). λ_{ex} = 351 nm.

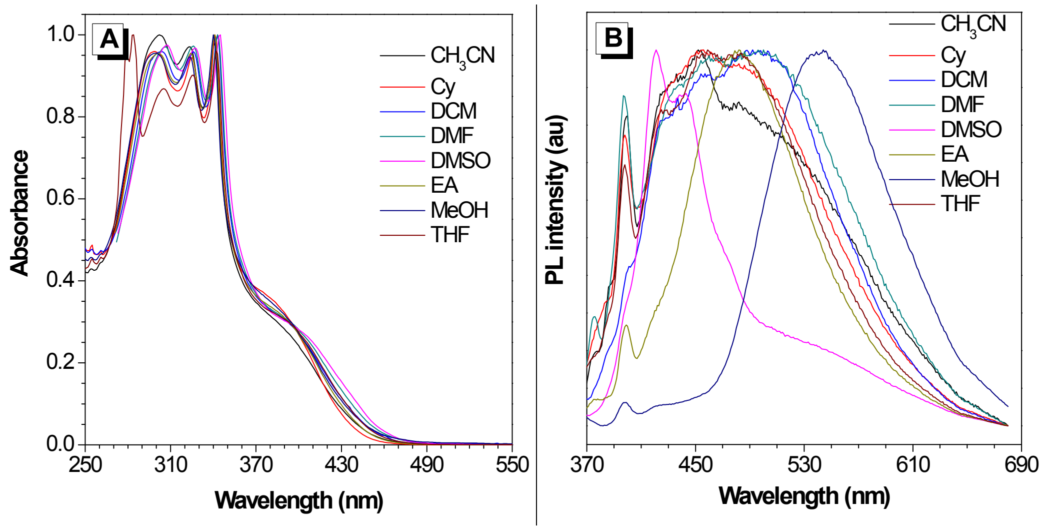

Fig. S14. (A) UV/Vis and (B) PL spectra of **2c** in different solvents (10 μM). λ_{ex} = 348 nm.

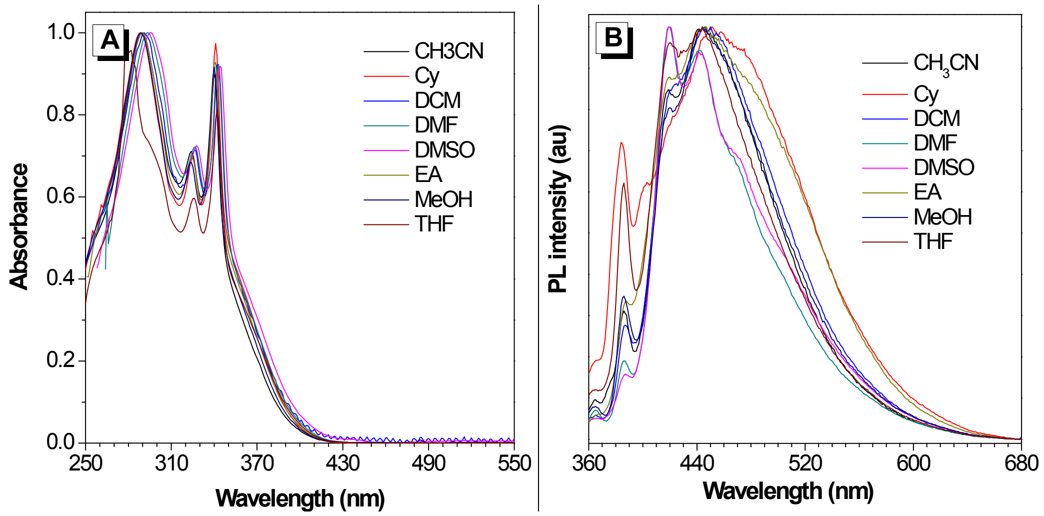

Fig. S15. (A) UV/Vis and (B) PL spectra of **3** in different solvents (10 μM). λ_{ex} = 347 nm.

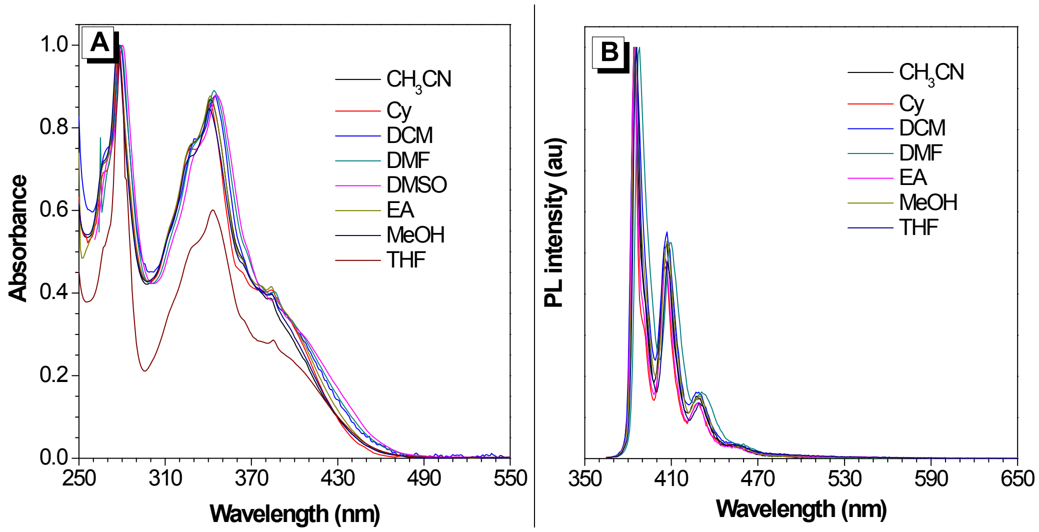

Figure S16. (A) UV/Vis and (B) PL spectra of **4** in different solvents (10 μM). λ_{ex} = 350 nm.

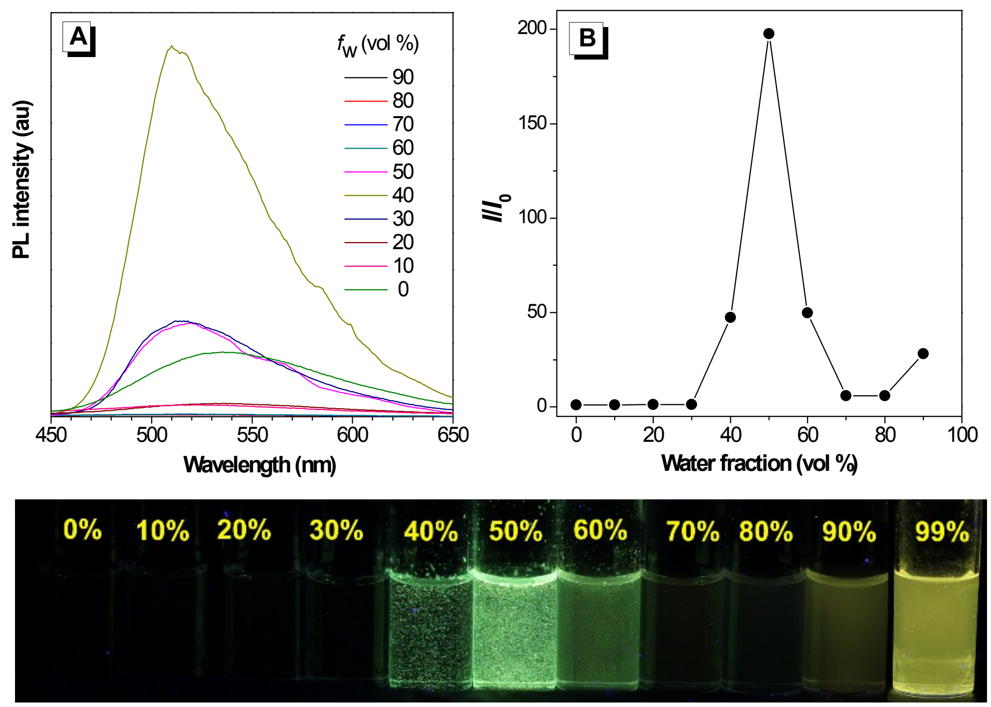

Fig. S17. (A) PL spectra of **2b** in THF/water mixtures with different water fractions (f_w). (B) Plot of relative PL intensity (I/I_0) versus the composition of THF/water mixtures of **2b**, where I_0 is the PL intensity in pure THF solution. Below: photographs in THF/water mixtures taken under UV illumination. Excitation wavelength: 365 nm.

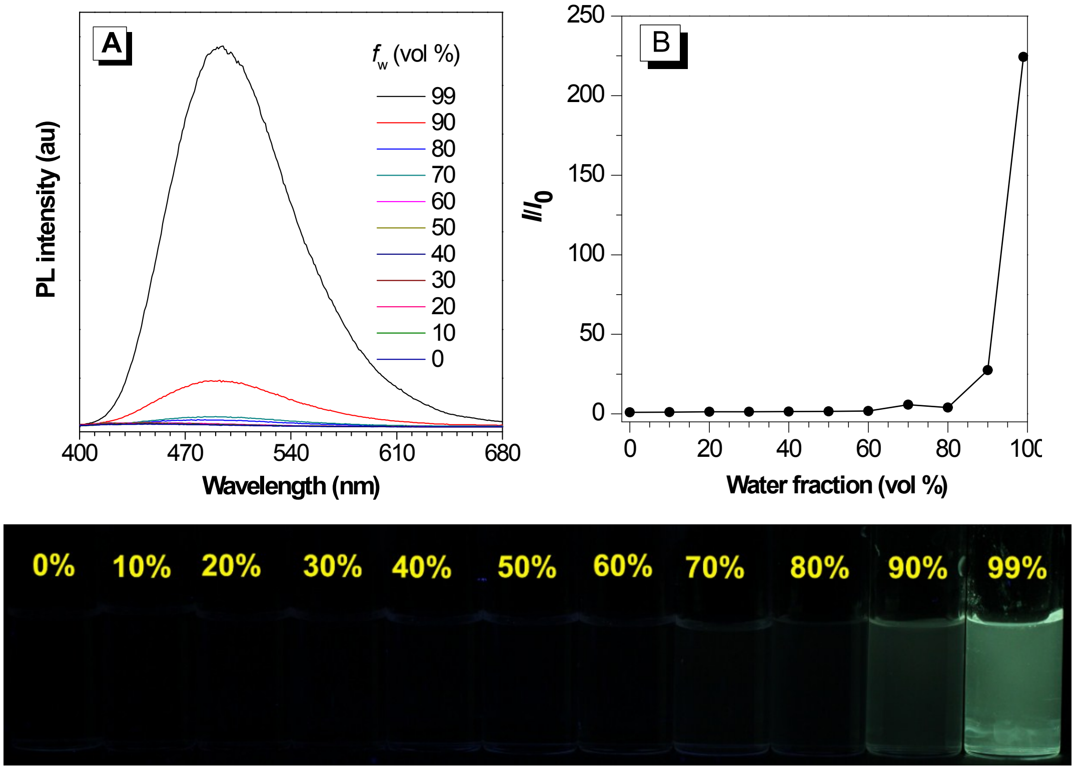

Fig. S18. (A) PL spectra of **3** in THF/water mixtures with different water fractions (f_w). (B) Plot of relative PL intensity (I/I_0) versus the composition of THF/water mixture of

3, where I_0 is the PL intensity in pure THF solution. Below: photographs in THF/water mixtures taken under UV illumination. Excitation wavelength: 365 nm.

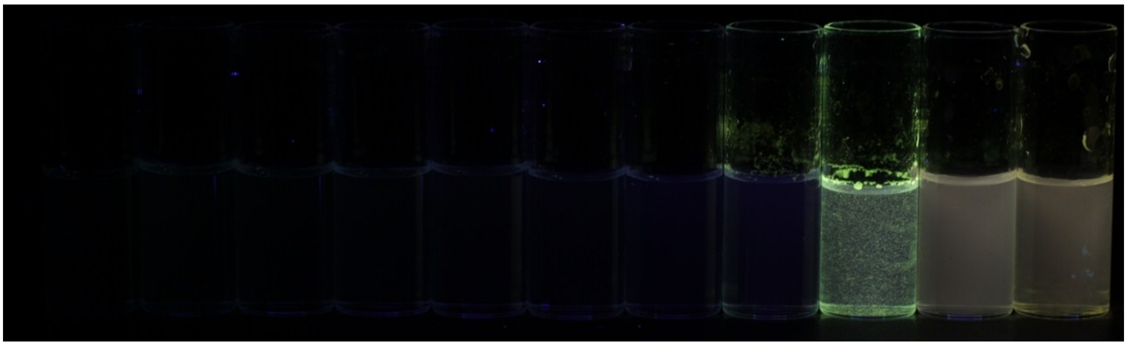

Fig. S19. Photographs of compound **2c** in THF/water mixtures taken under UV illumination. Excitation wavelength: 365 nm.

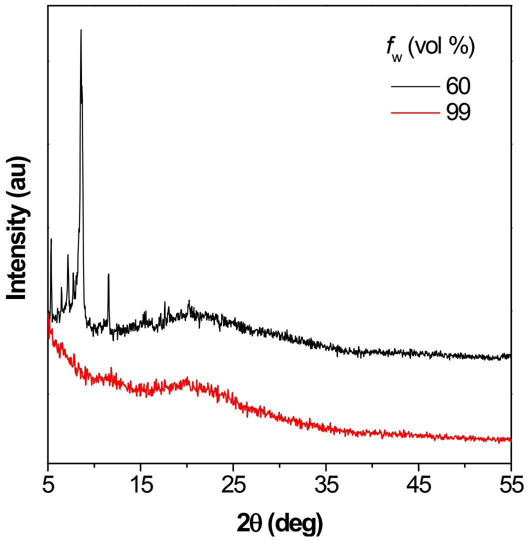

Figure S20. XRD diffractogram of **2a.**

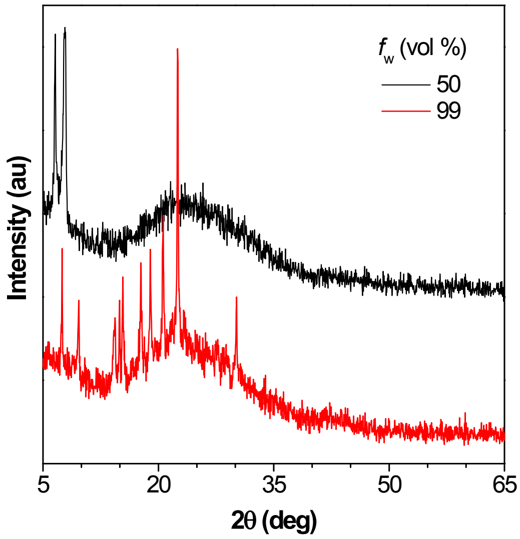

Fig. S21. XRG diffractogram of **2b.**

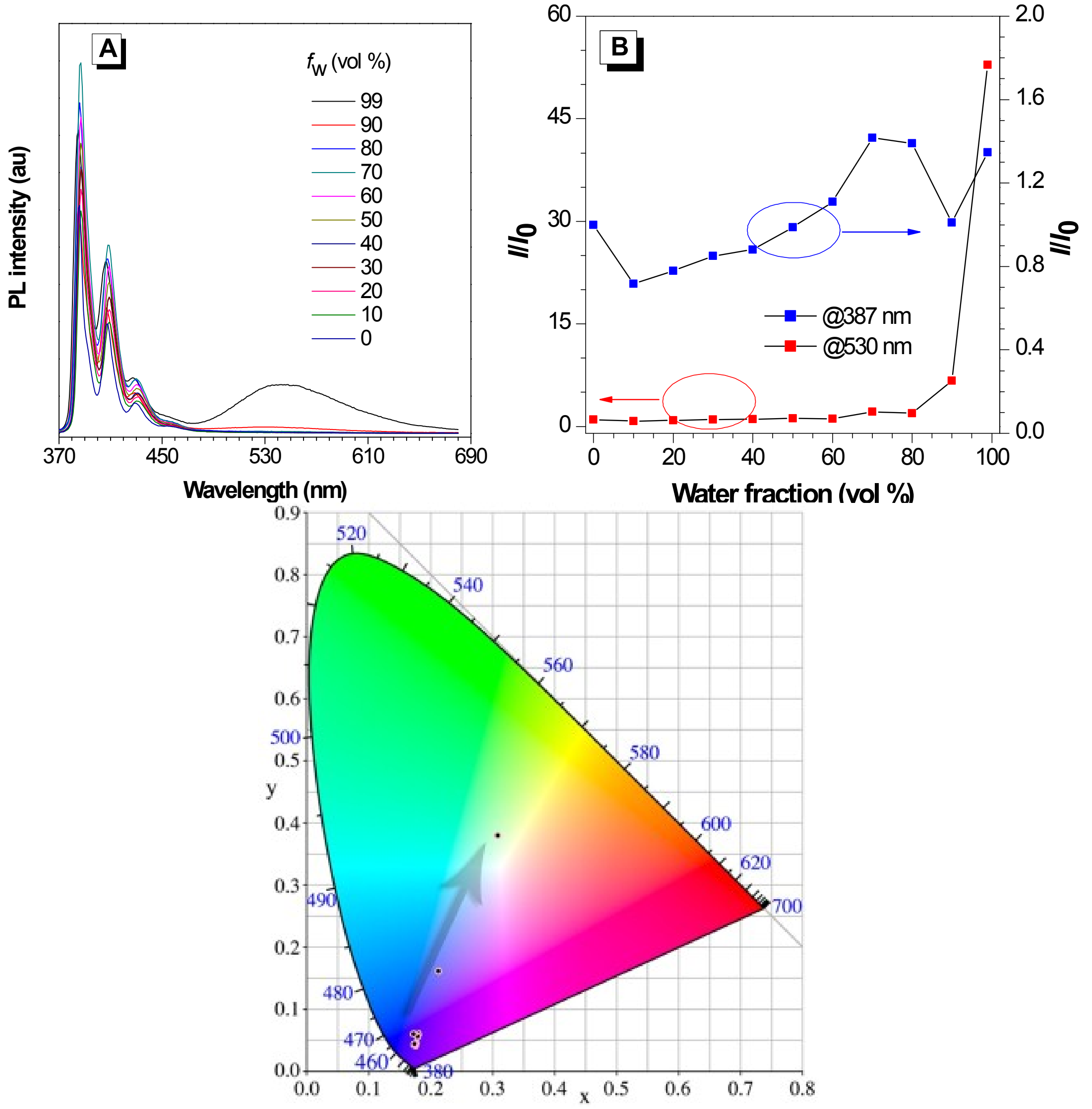

Figure S22. (A) PL spectra of **4** in THF/water mixtures with different water fractions (f_w). (B) Plots of relative PL intensity (I/I_0) versus the composition of THF/water mixtures of **4**, where I_0 is the PL intensity in pure THF solution. Inset: CIE chromaticity diagram of **4** in THF/water mixtures with different f_w (0–99 vol %).

Table S1. Optical properties of **2-4** in various solvents at room temp[a]

Cpd	$\lambda_{abs}/\lambda_{em}$ (nm)							
	CH_3CN	cyclohexane	CH_2Cl_2	DMF	DMSO	EA	MeOH	THF
**2a**	291, 341/454	286, 342/456	292, 344/456	295, 344/457	297, 345 /456	288, 342 /456	288, 341/456	294, 343/462
**2b**	--	298, 341/ 47	302, 343/449	304, 345/447, 508	306, 344 /421, 531	299, 341 /453	298, 340/451	304, 325, 394/500
**2c**	302, 324/452	299, 325/456	304, 325/492	306, 325/500	308, 328 /421, 443	301, 324 /482	299, 323/544	304, 342, 390/435
**3**	290, 324/446	288, 323/451	291, 327/450	293, 327/419, 442	296, 328 /419, 442	290, 324 /447	289, 324/441	279, 342/441
**4**	278, 341/384, 407, 428	278, 341/384, 406, 428	278, 345/385, 407, 429	279, 344/388, 409, 431		277, 341 /384, 406, 429	277, 341/385, 407, 430	278, 341, 400/386, 407, 429

[a] All measurements were performed under degassed conditions at a concentration of 10 μ M. Abbreviation: λ_{abs} = absorption maximum, λ_{em} = emission maximum, DMF = dimethylformamide, DMSO = dimethylsulfoxide, EA = ethyl acetate.

Table S2. The quantum yield of **2-4** in THF and mixture solvents of THF/H2O at room temp

**compound**	**Φ_F (%) in THF**	**Φ_F (%) In THF/H2O**	**Φ_F (%) in solid state**
**2a**	0.8	--	46.7
**2b**	0.6	6.7 (90%)	3.8
**2c**	0.6	12.0 (90%)	6.8
**3**	0.5	42.1 (90%)	19.8
**4**	0.8	22.1 (80%) / 28.8 (90%)	9.7

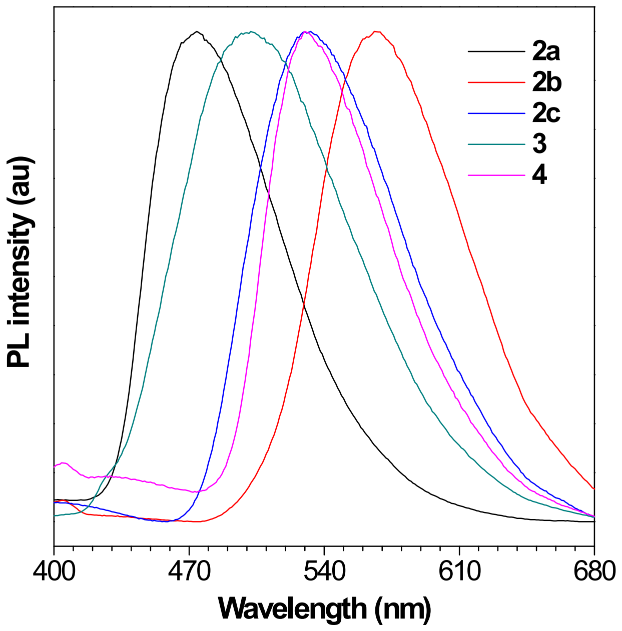

Figure S23. PL spectra of **2–4** in the solid state.

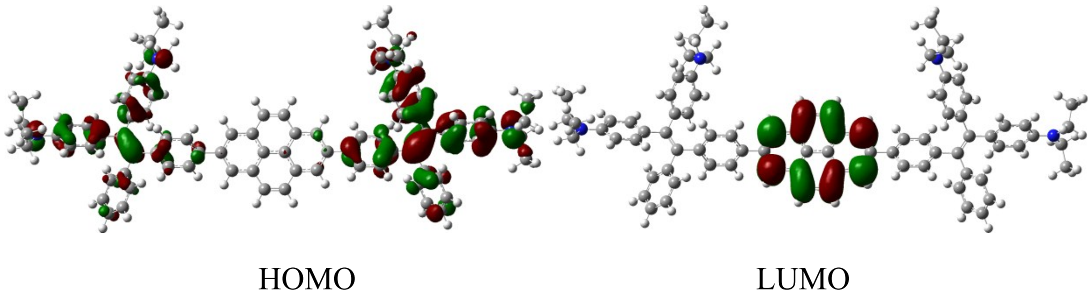

Figure S24. Computed molecular orbital plots of **2b** calculated by B3LYP/6–31G*.
